# Genetic overlap of chronic obstructive pulmonary disease and cardiovascular disease-related traits: a large-scale genome-wide cross-trait analysis

**DOI:** 10.1186/s12931-019-1036-8

**Published:** 2019-04-02

**Authors:** Zhaozhong Zhu, Xiaofang Wang, Xihao Li, Yifei Lin, Sipeng Shen, Cong-Lin Liu, Brain D. Hobbs, Kohei Hasegawa, Liming Liang, International COPD Genetics Consortium, H. Marike Boezen, Carlos A. Camargo, Michael H. Cho, David C. Christiani

**Affiliations:** 1 Department of Environmental Health, Harvard T.H. Chan School of Public Health, Boston, MA USA; 2 Program in Genetic Epidemiology and Statistical Genetics, Department of Epidemiology, Harvard T.H. Chan School of Public Health, Boston, MA USA; 3 Department of Cardiology, First Affiliated Hospital, College of Medicine, Zhengzhou University, Zhengzhou, China; 4 Department of Biostatistics, Harvard T.H. Chan School of Public Health, Boston, MA USA; 5 Department of Medicine, Brigham and Women’s Hospital and Harvard Medical School, Boston, MA USA; 6 Channing Division of Network Medicine, Brigham and Women’s Hospital, Boston, MA USA; 7 Department of Emergency Medicine, Massachusetts General Hospital, Boston, MA USA; 8 Department of Epidemiology, University Medical Center Groningen, University of Groningen, Groningen, the Netherlands; 9 Groningen Research Institute for Asthma and COPD, University Medical Center Groningen, University of Groningen, Groningen, the Netherlands; 10 Division of Pulmonary and Critical Care Medicine, Brigham and Women’s Hospital, Boston, MA USA; 11 Pulmonary and Critical Care Unit, Department of Medicine, Massachusetts General Hospital, Boston, MA USA

**Keywords:** Chronic obstructive pulmonary disease, Cardiovascular diseases, Genetic overlap

## Abstract

**Background:**

A growing number of studies clearly demonstrate a substantial association between chronic obstructive pulmonary disease (COPD) and cardiovascular diseases (CVD), although little is known about the shared genetics that contribute to this association.

**Methods:**

We conducted a large-scale cross-trait genome-wide association study to investigate genetic overlap between COPD (N_case_ = 12,550, N_control_ = 46,368) from the International COPD Genetics Consortium and four primary cardiac traits: resting heart rate (RHR) (*N* = 458,969), high blood pressure (HBP) (N_case_ = 144,793, N_control_ = 313,761), coronary artery disease (CAD)(N_case_ = 60,801, N_control_ = 123,504), and stroke (N_case_ = 40,585, N_control_ = 406,111) from UK Biobank, CARDIoGRAMplusC4D Consortium, and International Stroke Genetics Consortium data.

**Results:**

RHR and HBP had modest genetic correlation, and CAD had borderline evidence with COPD at a genome-wide level. We found evidence of local genetic correlation with particular regions of the genome. Cross-trait meta-analysis of COPD identified 21 loci jointly associated with RHR, 22 loci with HBP, and 3 loci with CAD. Functional analysis revealed that shared genes were enriched in smoking-related pathways and in cardiovascular, nervous, and immune system tissues. An examination of smoking-related genetic variants identified SNPs located in 15q25.1 region associated with cigarettes per day, with effects on RHR and CAD. A Mendelian randomization analysis showed a significant positive causal effect of COPD on RHR (causal estimate = 0.1374, *P* = 0.008).

**Conclusion:**

In a set of large-scale GWAS, we identify evidence of shared genetics between COPD and cardiac traits.

**Electronic supplementary material:**

The online version of this article (10.1186/s12931-019-1036-8) contains supplementary material, which is available to authorized users.

## Background

Chronic obstructive pulmonary disease (COPD) is a chronic inflammatory disease of the lungs that is the fourth leading cause of death in the world, accounting for more than 3 million deaths each year [1]. There is now considerable evidence of an association between COPD and cardiovascular disease (CVD). Several population-based studies have shown that COPD and airflow limitation is a predictor of cardiovascular risk [2]. The SUMMIT randomized clinical trial reported that exacerbations of COPD confer an increased risk of subsequent CVD [3, 4]. The Lung Health Study reported that for every 10% decrease in forced expiratory volume in 1 s (FEV1), there is a 28% increase in fatal coronary events among subjects with mild to moderate COPD [5]. In addition, CVD is a leading cause of death in patients with COPD, with a 5-year mortality of up to 25% due to a cardiovascular event [5, 6], such as high resting heart rate (RHR), systemic hypertension, coronary artery disease (CAD), or stroke [7–10].

We and colleagues recently identified shared genetic architecture between COPD and lung function/pulmonary fibrosis [11], asthma and allergic diseases [12], Alzheimer’s disease and metabolic disorders [13], psychiatric disorders [14], indicating potential pleiotropic effects among these diseases. COPD and CVD are both highly heritable traits [11, 15]. Parallel epidemic trends worldwide suggest shared genetic and environmental components for both conditions. However, there is little knowledge about shared genetic components between COPD and CVD. Although a previous study identified some genetic loci that influencing both lung function and CAD [16], the findings were not genome-wide in scale and were limited by small sample size. Therefore, it remains largely unknown to what extent the phenotypic association between COPD and CVD is due to shared genetic and biologic effects.

Therefore, we investigated the genetic correlation between COPD and cardiac traits and attempted to describe the specific shared genetic loci and biological pathways between traits. We conducted a large-scale, genome-wide association study (GWAS) cross-trait analysis of COPD from the International COPD Genetics Consortium (ICGC) and 4 cardiac traits from UK Biobank, CARDIoGRAMplusC4D Consortium, and International Stroke Genetics Consortium (ISGC) data, including RHR, high blood pressure (HBP), CAD [17], and stroke [18].

## Methods

### Study populations

**Fig. 1:**
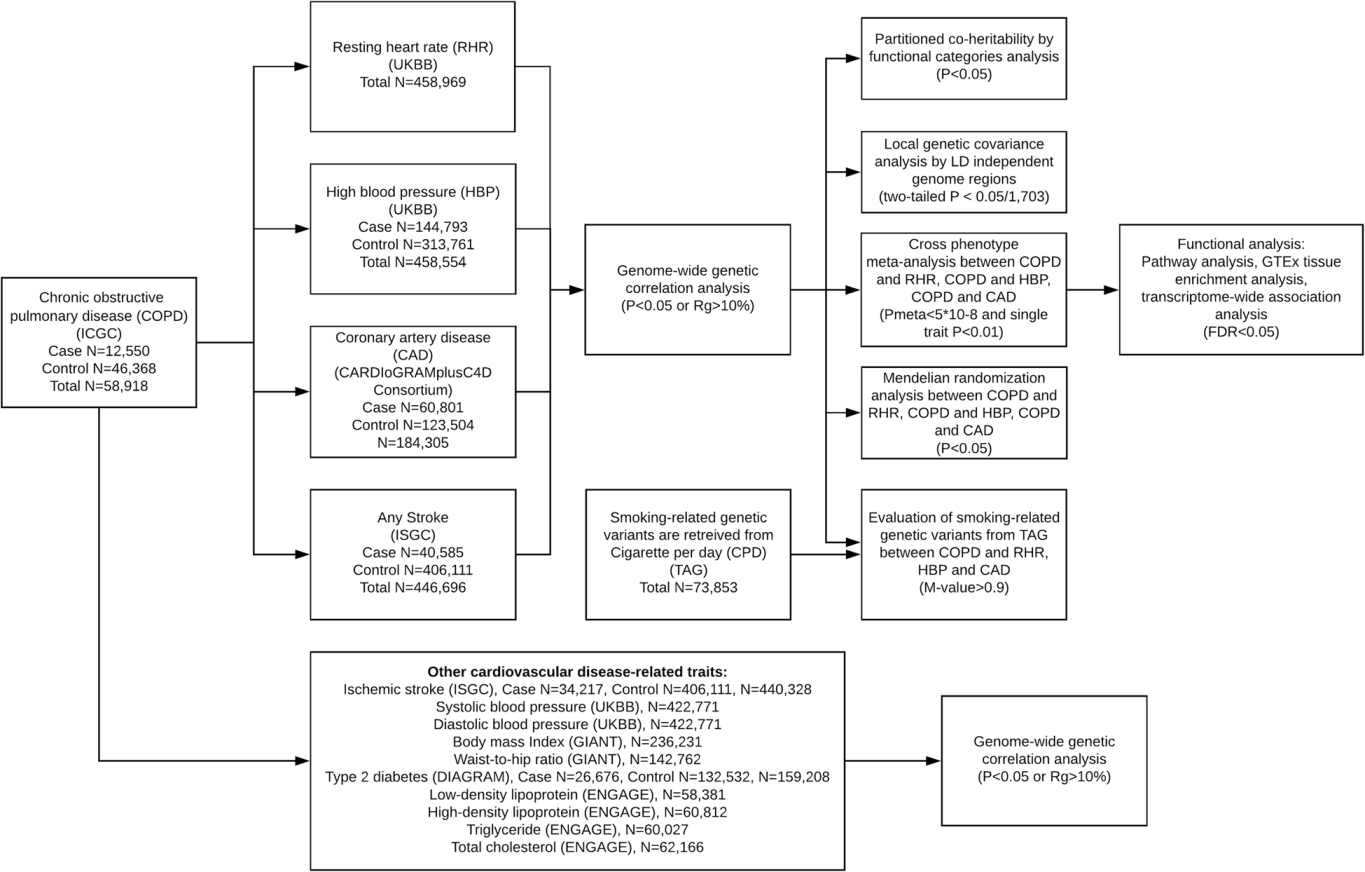
Overall study design. Multiple GWAS data sources were first retrieved. We first conducted genome-wide genetic correlation between COPD and 4 major cardiovascular disease (CVD) traits. For CVD traits that were shown genetic correlation with COPD, we conducted further post-GWAS analyses to investigate genetic overlap between them (variant/region/functional levels, smoking effect and causal inference). We also evaluated the genetic correlation between COPD and other CVD related traits. Abbreviations: ICGC: International COPD Genomic Consortium; UKBB: UK Biobank; ISGC: International Stroke Genetics Consortium; GIANT: The Genetic Investigation of ANthropometric Traits (GIANT) consortium; DIAGRAM: DIAbetes Genetics Replication And Meta-analysis consortium; ENGAGE: European Network for Genetic and Genomic Epidemiology consortium; TAG: Tobacco and Genetics Consortium

We included 4 major data sources—ICGC, UK Biobank, CARDIoGRAMplusC4D Consortium, and ISGC—in the overall study design (Fig. 1). Previous reports have detailed disease definition and baseline characteristics of the ICGC study cohorts [11] and UK Biobank cohort [19]. In brief, the ICGC defined COPD by GOLD criteria based on pre-bronchodilator spirometry: FEV1 of < 80% and FEV1 to forced vital capacity (FVC) ratio of < 0.7 for cases; or FEV1 of > 80% and FEV1/FVC of > 0.7 for controls, and adjusted for age, sex, pack-years, and smoking status. In UK Biobank, we used both data field 102 and 95 for RHR and data field 6150 for HBP. RHR was assessed via two methods: automated reading during blood pressure measurement (in 501,340 participants); and pulse waveform obtained from the finger with an infrared sensor during arterial stiffness measurement (in 193,472 participants). RHR was averaged if multiple measurements were available for one individual [20]. HBP was assessed by touch screen questionnaire of participants’ HBP diagnosis by doctor. We retrieved summary statistics from publicly available GWAS studies: CAD (N_case/control_ = 60,801/123,504) from CARDIoGRAMplusC4D Consortium [17], and stroke (N_case/control_ = 40,585/406,111) from ISGC [18]. CAD diagnoses in CARDIoGRAMplusC4D was defined by an inclusive CAD diagnosis (e.g. myocardial infarction (MI), acute coronary syndrome, chronic stable angina, or coronary stenosis > 50%) [17]. The ISGC defined stroke by an inclusive stroke diagnosis (e.g. ischemic stroke, large artery stroke, cardioembolic stroke and small vessel stroke). We standardized GWAS summary data to minimize potential bias due to quality control procedures. Indels and rare/low frequency variants with a minor allele frequency of < 1% were excluded. In addition, we restricted analysis to autosomal chromosomes. Aside from RHR and HBP, both tested in Biobank, we are not aware of specific sample overlap between COPD and 4 major cardiovascular traits in this study, including RHR, HBP, CAD and stroke. Details of each dataset can be found in Additional file 1: Table S1. All subjects consent to participate the study by the time of data analysis.

### GWAS analysis in UK biobank

We performed GWAS analysis on RHR and HBP using a linear mixed model (LMM) method [21] based on European ancestry. See the Additional file 2: Supplemental Note for additional information.

### LD score regression (LDSC) analysis

We conducted post-GWAS genetic correlation analysis with LDSC, which estimates genetic correlation between true causal effects of two traits (genetic correlation estimate Rg ranging from − 1 to 1) [22]. Cardiac traits showing genome-wide genetic correlation with COPD were further studied in the downstream analysis. See the Additional file 2: Supplemental Note for additional information.

In addition, we performed genetic correlation analysis between COPD and ischemic stroke subtypes, and metabolic traits (lipids, obesity, and glucose).

### Partitioned genetic correlation

To characterize genetic overlap at the level of functional categories, we estimated genetic correlation between COPD and cardiac traits in 11 annotation categories using LDSC. These annotations included transcribed regions, transcription factor binding sites, super-enhancers, introns, DNaseI digital genomic footprinting (DGF) regions, DNaseI hypersensitivity sites (DHSs), fetalDHSs, and histone marks h3k9ac, h3k4me1, h3k4me3, and h3k27ac [23]. For each annotation, we re-calculated LD scores for SNPs assigned to that particular category and then used annotation-specific LD scores to estimate the COPD–cardiac trait genetic correlation.

### Local genetic correlation

To identify local genetic correlations between COPD and cardiac traits, we performed ρ-HESS to estimate local genetic correlation between a pair of traits at each LD-independent region in the genome [24]. Approximately 1703 independent LD blocks of 1.5 Mb were used to calculate local genetic heritability and covariance. All GWAS data were restricted to European ancestry, and Bonferroni correction was used to adjust multiple testing (two-tailed *P* < 0.05/1703) according to the original method description [24].

### Cross-trait meta-analysis

After assessing genetic correlations among all traits, we applied 2 cross-trait GWAS meta-analysis methods to combine binary or continuous traits [25]. We used association analysis based on SubSETs (ASSET) to combine association evidence for COPD with HBP and CAD at individual variants because it is designed for meta-analysis of binary traits [26]. We also applied another cross-trait GWAS meta-analysis method, cross phenotype association (CPASSOC), to combine association evidence for COPD with RHR at individual variants, since this method allows meta-analysis of continuous traits [27]. See the Additional file 2: Supplemental Note for additional information.

We applied PLINK [28] clumping function (parameters: --clump-p1 5e-8 --clump-p2 1e-5 --clump-r2 0.2 --clump-kb 500) to determine top loci that were independent from one another (i.e., variants with *P* < 1 × 10^− 5^, r^2^ > 0.2, and < 500 kb away from a peak). The variant with the lowest *p*-value was defined as the sentinel variant. Putative genes for each variant were considered to be those within the clump. We used Variant Effect Predictor based on Ensembl/GENCODE basic transcripts database for detailed variant annotation [29].

### Fine-mapping of credible sets

To identify the 99% credible set of variants within each 500-kb sentinel variant, we identified a credible set of causal variants at each shared locus that met cross-trait meta-analysis criteria using the Bayesian likelihood fine-mapping algorithm [30]. The algorithm maps primary signal and uses a flat prior with steepest descent approximation.

### Pathway and GTEx tissue enrichment analysis

To gain biological insights for shared genes, we used the WebGestalt tool [31] to assess enrichment of the identified shared gene set in the Gene Ontology (GO) biological process. We conducted GTEx tissue enrichment analysis using functional mapping and annotation (FUMA) [32] with 53 tissue types from GTEx version 7 [33]. Both analyses were based on shared genes that were identified from cross-trait meta-analysis.

### Transcriptome-wide association study (TWAS)

To identify shared COPD and cardiac trait gene expression associations in specific tissues, we conducted TWAS using the FUSION software package based on 43 GTEx (version 6) tissue expression weights [34]. Multiple testing correction was applied for each trait’s gene–tissue pairs on TWAS *P*-values using false discovery rate (FDR) Benjamini-Hochberg procedure (FDR < 0.05).

### Evaluation of effect of smoking-related genetic variants between COPD and cardiac traits

To evaluate the potential effect of smoking-related genetic variants between COPD and cardiac traits, we retrieved 129 genome-wide significant SNPs for cigarette per day (CPD) from the Tobacco and Genetics Consortium (TAG) [35]. We also looked up GWAS results for 2 other smoking related traits from TAG, ever vs never smoked and current vs former smoker, however no SNPs reached genome-wide significance. Thus, we merged 129 SNPs with COPD and CVD traits (RHR, HBP and CAD) and identified 45 SNPs in common for all traits. We used M-value posterior probability [36] to evaluate if the CPD genetic variant effect exists among COPD and CVD traits. A M-value > 0.9 was considered evidence that the SNP had an effect on the trait.

### Mendelian randomization (MR) analysis

Finally, we performed MR analysis using Mendelian Randomization Pleiotropy RESidual Sum and Outlier (MR-PRESSO) [37] in order to infer putative causal relationships between COPD and 3 cardiac traits (RHR, HBP, CAD). MR-PRESSO estimates effect of exposure on outcome using SNPs significantly associated with exposure and allows for the evaluation of horizontal pleiotropy in multi-instrument Mendelian Randomization utilizing GWAS summary association statistics. We constructed instruments using genome-wide significant LD-independent SNPs with *P*-value less than 5 × 10^− 8^. Prior to running MR-PRESSO, we removed strand-ambiguous SNPs and SNPs in the MHC region (chr6:25-34 M).

## Results

### Genome-wide association and SNP-based heritability

The phenotype–genotype association test was carried out on ~ 460,000 samples and ~ 5.26 million SNPs from UK Biobank data after quality control. The genomic inflation factor (λ_gc_) from LDSC for RHR and HBP were 1.8405 [LDSC intercept: 1.1256, standard error (SE): 0.0502; Additional file 3: Figure S1] and 1.7648 (LDSC intercept: 1.1061, SE: 0.0244; Additional file 3: Figure S2), respectively; these values suggest that much of the inflation is due to polygenic inheritance [38]. Estimates of SNP-based heritability on the observed scale using GWAS summary statistics were 20.11% (SE: 2.61%) for COPD, 15.19% (SE: 1.28%) for RHR, 12.80% (SE: 0.58%) for HBP, 6.71% (SE: 0.52%) for CAD, and 1.21% (SE: 0.14%) for stroke (Additional file 1: Table S2).

### Genome-wide genetic correlation

We evaluated the genetic correlation of COPD and cardiac traits using cross-trait LDSC. Nominally significant genetic correlation with COPD was found for both RHR (Rg = 0.0722; *P* = 0.0434) and HBP (Rg = 0.0751; *P* = 0.0467) (Table 1). Genetic correlation for COPD and CAD was approximately 10%, but this value did not reach statistical significance; we did not observe significant genetic correlation between COPD and stroke (Table 1), or additional blood pressure traits, such as systolic blood pressure, diastolic blood pressure (Additional file 1: Table S3). In addition, we did not find evidence of genetic correlation between COPD and ischemic stroke subtype or any CVD related metabolic traits (Additional file 1: Table S3).

**Table 1:** Genome-wide genetic correlation between COPD and cardiac traits

Phenotype 1	Phenotype 2	Rg	Rg_SE	*P*
COPD	Resting heart rate	0.0722	0.0357	0.0434
COPD	High blood pressure	0.0751	0.0378	0.0467
COPD	Coronary artery disease	0.1015	0.0528	0.0548
COPD	Stroke	0.0226	0.0689	0.7428

*COPD* chronic obstructive pulmonary disease, *Rg* genetic correlation estimate, *SE* standard error

### Partitioned genetic correlation

In partitioned LDSC analysis, we used 11 functional annotations to evaluate genetic correlations between COPD and cardiac traits by specific functional category. The highest magnitude of significant genetic correlation between COPD and HBP was in introns (Rg = 0.1711; *P* = 0.0233) and h3k9ac (Rg = 0.1428; *P* = 0.033) (Additional file 3: Figure S3, Additional file 1: Table S4). Super enhancers had the highest magnitude of genetic correlation between COPD and RHR (Rg = 0.1259; *P* = 0.0173).

### Local genetic correlation

**Fig. 2:**
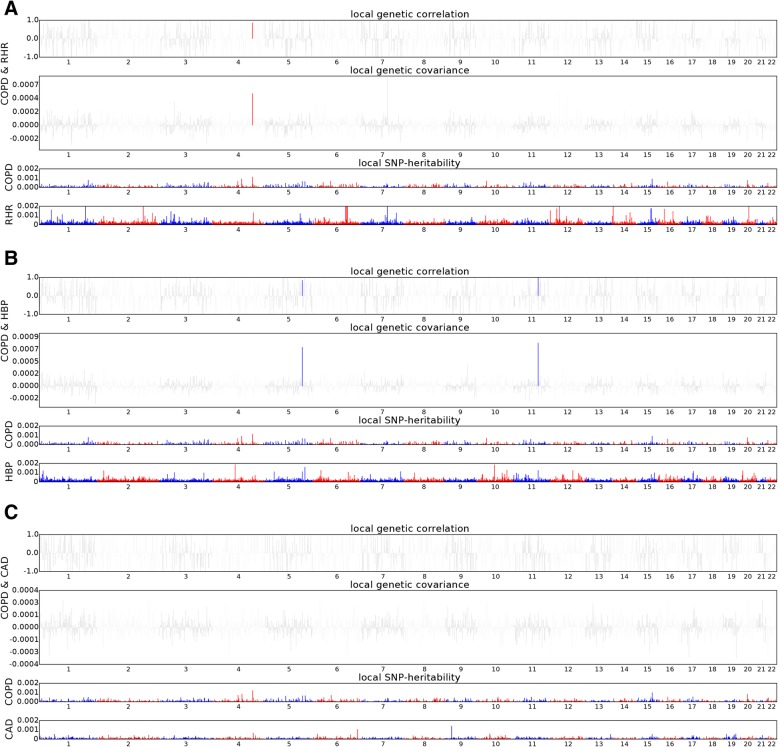
Plots depicting local genetic correlation (top), genetic covariance (middle), and SNP heritability (bottom) for COPD and RHR (**a**), COPD and HBP (**b**), and COPD and CAD (**c**). Blue or red highlights indicate significant local genetic correlation and covariance after multiple testing correction

We performed *ρ*-HESS to investigate whether specific regions of the genome had genetic correlation between COPD and cardiac traits. Analysis of the COPD/RHR trait pair showed that the 4q31 region (chromosome 4: 143443265–146,178,187) had strong local genetic correlations (*P* = 7.42 × 10^− 7^) (Fig. 2 and Additional file 1: Table S5). Analysis of the COPD/HBP trait pair showed strong local genetic correlations in 11q22 (chromosome 11: 100417169–101,331,121; *P* = 6.31 × 10^− 7^) and 5q32 (chromosome 5: 147181998–148,662,624; *P* = 3.98 × 10^− 6^) regions (Fig. 2 and Additional file 1: Table S6). We did not observe any significant local genetic correlations for the COPD/CAD trait pair (Fig. 2 and Additional file 1: Table S7).

### Cross-trait meta-analysis between COPD and cardiac traits

ASSET and CPASSOC were applied for genome-wide meta-analysis to identify genetic loci associated with COPD and cardiac traits (meta-analysis *P* < 5 × 10^− 8^; trait-specific *P* < 0.01). After pruning, we found 21 loci significantly associated with COPD and RHR (Table 2 and Additional file 1: Table S8). The most significant SNP was rs7655625 *(P*_*meta*_ = 1.92 × 10^− 19^), located at the *HHIP* locus. The second most significant locus (sentinel SNP: rs59985166, *P*_*meta*_ = 4.08 × 10^− 18^) was located at the *CASZ1* locus [39].

**Table 2:** Genome-wide significant loci by cross-trait meta-analysis at sentinel SNPs associated with COPD and RHR (*P*_meta_ < 5 × 10^− 8^; single trait *P* < 0.01)

Sentinel SNP	CHR	N	Position	*P*_RHR_	*P*_COPD_	*P*_META_	Variant annotation	Genes within clumping region
rs3738676	1	162	chr1:39552963–40,088,043	2.30 × 10^− 10^	1.51 × 10^− 3^	3.07 × 10^− 11^	3′ UTR	*BMP8A, KIAA0754, MACF1, PABPC4, PPIEL, SNORA55*
rs59985166	1	47	chr1:10653622–10,753,094	2.50 × 10^−16^	2.92 × 10^− 3^	4.08 × 10^−18^	Intron	*CASZ1, PEX14*
rs13409705	2	17	chr2:28820641–29,115,712	2.80 × 10^− 10^	6.57 × 10^− 4^	8.61 × 10^− 12^	Intron	*PLB1, PPP1CB, SPDYA, TRMT61B*
rs3791979	2	21	chr2:218667372–218,704,894	2.40 × 10^− 9^	4.15 × 10^− 3^	2.0 × 10^− 9^	3′ UTR	*TNS1*
rs2955083	3	119	chr3:127715196–128,067,275	6.90 × 10^− 4^	2.0 × 10^−8^	3.56 × 10^− 8^	Intron	*EEFSEC, RUVBL1, SEC61A1*
rs9859058	3	78	chr3:156353161–156,695,011	3.60 × 10^−10^	6.57 × 10^−3^	4.01 × 10^− 11^	Intron	*LEKR1, LINC00886, PA2G4P4, TIPARP, TIPARP-AS1*
rs7655625	4	140	chr4:145196359–145,974,688	2.70 × 10^−5^	3.02 × 10^−19^	1.92 × 10^− 19^	Intergenic	*ANAPC10, HHIP, HHIP-AS1*
rs7449334	5	43	chr5:90309787–90,418,617	1.0 × 10^− 10^	3.59 × 10^− 3^	1.62 × 10^−11^	Intron	*GPR98*
rs1158317	6	5	chr6:120518685–121,035,654	6.50 × 10^−11^	7.45 × 10^−3^	1.19 × 10^− 11^	Intergenic	C6orf170*
rs12173787	6	144	chr6:33350201–33,789,899	1.20 × 10^−15^	5.4 × 10^−3^	2.79 × 10^− 15^	Downstream gene	*BAK1, CUTA, GGNBP1, IP6K3, ITPR3, KIFC1, LEMD2, LINC00336, MIR5004, MLN, PHF1, SYNGAP1, UQCC2, ZBTB9*
rs57942103	8	11	chr8:106513461–106,589,409	1.90 × 10^−10^	5.06 × 10^−3^	3.10 × 10^− 11^	Intron	*ZFPM2*
rs10883944	10	21	chr10:105522875–105,667,110	7.80 × 10^−9^	2.11 × 10^−4^	2.33 × 10^−10^	Intron	*OBFC1, SH3PXD2A*
rs4746139	10	92	chr10:75404300–75,692,923	4.50 × 10^−10^	9.94 × 10^−3^	1.60 × 10^− 10^	Synonymous	*AGAP5, BMS1P4, C10orf55, CAMK2G, CHCHD1, FUT11, GLUD1P3, NDST2, PLAU, SEC24C, SYNPO2L, ZSWIM8, ZSWIM8-AS1*
rs2512519	11	68	chr11:77924870–78,286,462	2.10 × 10^− 10^	2.32 × 10^−3^	4.39 × 10^−12^	Intron	*GAB2, NARS2, USP35*
rs8756	12	12	chr12:66306441–66,389,968	2.20 × 10^−11^	3.22 × 10^−3^	2.57 × 10^− 12^	3′ UTR	*HMGA2*
rs56386186	14	77	chr14:102418380–102,784,274	1.70 × 10^−10^	2.79 × 10^−3^	1.22 × 10^−11^	Intron	*DYNC1H1, HSP90AA1, MOK, WDR20, ZNF839*
rs7143026	14	17	chr14:65825854–66,272,664	1.90 × 10^−6^	1.22 × 10^−4^	2.54 × 10^−8^	Intergenic	*FUT8, FUT8-AS1*
rs754388	14	34	chr14:93068516–93,118,229	3.0 × 10^−4^	7.07 × 10^−12^	1.03 × 10^−11^	Intron	*RIN3*
rs17186681	15	138	chr15:63800237–64,148,582	2.50 × 10^−11^	6.57 × 10^−3^	1.50 × 10^−12^	Intron	*FBXL22, HERC1, USP3, USP3-AS1*
rs17431820	16	10	chr16:64939145–65,128,819	5.30 × 10^−12^	9.78 × 10^−3^	2.38 × 10^− 12^	Intron	*CDH11*
rs12709669	18	14	chr18:19780858–19,826,742	4.10 × 10^−9^	4.80 × 10^−3^	8.11 × 10^−10^	Intron	*GATA6*

*SNP* single nucleotide polymorphisms, *CHR* chromosome, *N* number of SNPs clumped with peak variant, *RHR* resting heart rate, *COPD* chronic obstructive pulmonary disease

In addition, we found 22 loci significantly associated with both COPD and HBP (Table 3 and Additional file 1: Table S9). The top significant locus was near *ARHGAP42* (sentinel SNP: rs633185, *P*_*meta*_ = 1.80 × 10^− 47^) [40], Notably, rs7655625, the most significant SNP for COPH/RHR, also had strong correlation with COPD/HBP *(P*_*meta*_ = 9.69 × 10^− 19^).

**Table 3:** Genome-wide significant loci by cross-trait meta-analysis at sentinel SNPs associated with COPD and HBP (*P*_meta_ < 5 × 10^− 8^; single trait *P* < 0.01)

Sentinel SNP	CHR	N	Position	*P*_HBP_	*P*_COPD_	*P*_META_	Variant annotation	Genes within clumping region
rs12759054	1	72	chr1:234091759–234,196,884	1.2 × 10^−6^	5.78 × 10^−3^	4.88 × 10^−8^	Intron	*SLC35F3*
rs301819	1	99	chr1:8440643–8,895,970	1.3 × 10^−10^	5.47 × 10^−3^	9.93 × 10^−12^	Intron	*RERE*
rs305221	1	115	chr1:88899115–89,440,896	3.7 × 10^−10^	5.32 × 10^−4^	2.91 × 10^−12^	Intron	*CCBL2, GTF2B, LOC101927891, PKN2*
rs61781370	1	97	chr1:39554034–40,060,025	9.0 × 10^−8^	6.66 × 10^−3^	7.47 × 10^−9^	Upstream gene	*BMP8A, KIAA0754, MACF1, PABPC4, PPIEL, SNORA55*
rs71636784	1	198	chr1:26994245**–**27,298,564	3.60 × 10^−10^	0.002705	6.41 × 10^−12^	Intron	*ARID1A, GPATCH3, GPN2, KDF1, NR0B2, NUDC, PIGV, SFN, ZDHHC18*
rs2293947	3	153	chr3:127620467–128,349,376	1.0 × 10^−8^	1.96 × 10^−3^	9.09 × 10^−10^	Intron	*C3orf27, DNAJB8, DNAJB8-AS1, EEFSEC, GATA2, KBTBD12, LOC90246, RPN1, RUVBL1, SEC61A1*
rs6799272	3	163	chr3:157977793–158,421,824	2.0 × 10^−8^	2.55 × 10^−3^	1.22 × 10^−9^	Intron	*GFM1, LOC100996447, LXN, MLF1, RARRES1, RSRC1*
rs2869966	4	135	chr4:89750361–90,028,653	2.20 × 10^−3^	1.11 × 10^−14^	2.73 × 10^−14^	Intron	*FAM13A*
rs7655625	4	111	chr4:145228728–145,974,688	7.10 × 10^−3^	3.02 × 10^−19^	9.69 × 10^−19^	Intergenic	*ANAPC10, HHIP, HHIP-AS1*
rs7733088	5	66	chr5:147682118–147,856,522	1.50 × 10^−3^	4.41 × 10^−14^	1.26 × 10^−13^	Intron	*FBXO38, HTR4, LOC102546294, SPINK7, SPINK9*
rs9399401	6	15	chr6:142652344–142,865,106	4.20 × 10^−3^	3.59 × 10^−10^	9.38 × 10^−10^	Intron	*GPR126, LOC153910*
rs11771259	7	73	chr7:7174042–7,348,633	1.60 × 10^−15^	6.16 × 10^−4^	6.66 × 10^−18^	Intron	*C1GALT1, LOC101927354*
rs36044436	7	40	chr7:74027839–74,140,925	7.40 × 10^−10^	3.77 × 10^−3^	4.87 × 10^−11^	Intron	*GTF2I, LOC101926943*
rs633185	11	256	chr11:100421331–100,713,227	1.90 × 10^−48^	1.63 × 10^− 4^	1.80 × 10^− 47^	Intron	*ARHGAP42*
rs11168245	12	125	chr12:47981942–48,212,719	1.20 × 10^−9^	6.23 × 10^−3^	7.26 × 10^−11^	Intron	*ENDOU, HDAC7, RAPGEF3, RPAP3, SLC48A1*
rs1549306	16	95	chr16:75304623–75,491,327	4.0 × 10^−9^	7.61 × 10^−4^	3.36 × 10^−11^	Intron	*CFDP1, TMEM170A*
rs200528	16	58	chr16:24699511–24,879,963	1.70 × 10^−7^	1.86 × 10^−3^	1.37 × 10^−8^	Intron	*SLC5A11, TNRC6A*
rs4787486	16	26	chr16:29958216–30,093,779	1.10 × 10^−8^	8.61 × 10^−3^	7.10 × 10^−9^	Intron	*ALDOA, C16orf92, DOC2A, FAM57B, HIRIP3, INO80E, PPP4C, TAOK2, TMEM219*
rs55804009	18	72	chr18:1840658–1,902,417	9.0 × 10^−9^	7.36 × 10^−3^	6.62 × 10^−10^	Intergenic	LINC00470*
rs13040716	20	44	chr20:30660621–31,035,129	1.20 × 10^−5^	3.36 × 10^−5^	4.0 × 10^−8^	Downstream gene	*ASXL1, HCK, KIF3B, NOL4L, PLAGL2, POFUT1, TM9SF4, TSPY26P*
rs12627514	21	29	chr21:44740327–44,824,964	1.70 × 10^−12^	7.55 × 10^−3^	2.82 × 10^−14^	Intergenic	*LINC00322*
rs229340	21	173	chr21:44914815–45,133,868	6.10 × 10^−11^	9.87 × 10^−3^	1.57 × 10^−11^	Intron	*HSF2BP, MIR6070, RRP1B*

*SNP* single nucleotide polymorphisms, *CHR* chromosome, *HBP* high blood pressure, COPD chronic obstructive pulmonary disease

In addition to rs7655625 in *HHIP*, we also observed two more overlapping significant loci in meta-analyses of COPD/RHR and COPD/HBP. The first locus was *EEFSEC* (sentinel SNP: rs2955083, *P*_*meta*_ = 3.56 × 10^− 8^ for COPD/RHR; sentinel SNP: rs2293947, *P*_*meta*_ = 9.09 × 10^− 10^ for COPD/HBP) [11, 41]. The other locus was *BMP8A* (sentinel SNP: rs3738676, *P*_*meta*_ = 3.07 × 10^− 11^ for COPD/RHR; sentinel SNP: rs61781370, *P*_*meta*_ = 7.47 × 10^− 9^ for COPD/HBP) [42, 43]. Finally, we identified 3 loci significantly associated with COPD and CAD (Table 4 and Additional file 1: Table S10). The first locus (sentinel SNP: rs2128739, *P*_*meta*_ = 3.17 × 10^− 12^) is a transcript of long non-coding RNA gene *RP11-563P16.1*. The second locus represented by rs8108474 (*P*_*meta*_ = 1.49 × 10^− 8^) was mapped to *DMPK* [44]. The third locus (sentinel SNP: rs8046697, *P*_*meta*_ = 3.80 × 10^− 8^), was mapped to *BCAR1* [45]. Detailed annotation for each sentinel variant is shown in Additional file 1: Table S11.

**Table 4:** Genome-wide significant loci by cross-trait meta-analysis at sentinel SNPs associated with COPD and CAD (*P*_meta_ < 5 × 10^−8^; single trait *P* < 0.01)

Sentinel SNP	CHR	N	Position	*P*_CAD_	*P*_COPD_	*P*_META_	Variant annotation	Genes within clumping region
rs2128739	11	13	chr11:103660567–103,718,660	7.05 × 10^−11^	3.69 × 10^−3^	3.17 × 10^−12^	Intergenic	*RP11-563P16.1*
rs8046697	16	164	chr16:75236763–75,516,534	3.24 × 10^−6^	8.1 × 10^−4^	3.80 × 10^−8^	Intron	*BCAR1, CFDP1, CHST6, CTRB1, CTRB2, LOC100506281, TMEM170A*
rs8108474	19	27	chr19:46190268–46,370,381	7.51 × 10^−6^	5.62 × 10^−5^	1.49 × 10^−8^	Intron	*DMPK, DMWD, FBXO46, FOXA3, LOC388553, QPCTL, RSPH6A, SIX5, SNRPD2, SYMPK*

*SNP* single nucleotide polymorphisms, *CHR* chromosome, *CAD* coronary artery disease, *COPD* chronic obstructive pulmonary disease

### Identification of causal variants

We identified a credible set of causal SNPs using Bayesian fine-mapping at each shared loci meeting significance criteria in the COPD–cardiac traits meta-analysis. The credible set of variants at each locus were 99% likely to contain the causal variant. A list of credible sets of SNPs for each locus is provided in Additional file 1: Tables S11–S14.

We found 5 loci (in *MACF1, SYNPO2L*, *RIN3*, *TNS1,* and *MLN*) for COPD/RHR (Additional file 1: Table S15), 4 loci (*NR0B2*, *C1orf172*, *MAFC1,* and *TNRC6A*) for COPD/HBP (Additional file 1: Table S16), and 7 loci (*CD3EAP*, *C19orf83*, *GIPR, FBXO46, AC074212.3, SIX5,* and *DMPK*) for COPD/CAD (Additional file 1: Table S17) in which the credible set included exonic missense polymorphisms. However, most variants in credible sets at each locus were either intronic or intergenic, which is consistent with prior studies showing most variants detected by GWAS involve gene regulatory effects, rather than protein structure changes [46].

### Biological pathway, tissue enrichment, and TWAS

We performed pathway analyses to identify biological pathways enriched for shared loci related to COPD and cardiac traits based on significant cross-trait meta-analysis results. COPD and RHR response to nicotine was present only at a liberal FDR (FDR = 0.198) (Additional file 1: Table S18). COPD shared pathways of detection of chemical stimulus involved in sensory perception of smell with HBP (FDR = 1.06 × 10^− 10^) (Additional file 1: Table S19). No biological pathways were significantly shared by COPD and CAD (Additional file 1: Table S20).

**Fig. 3:**
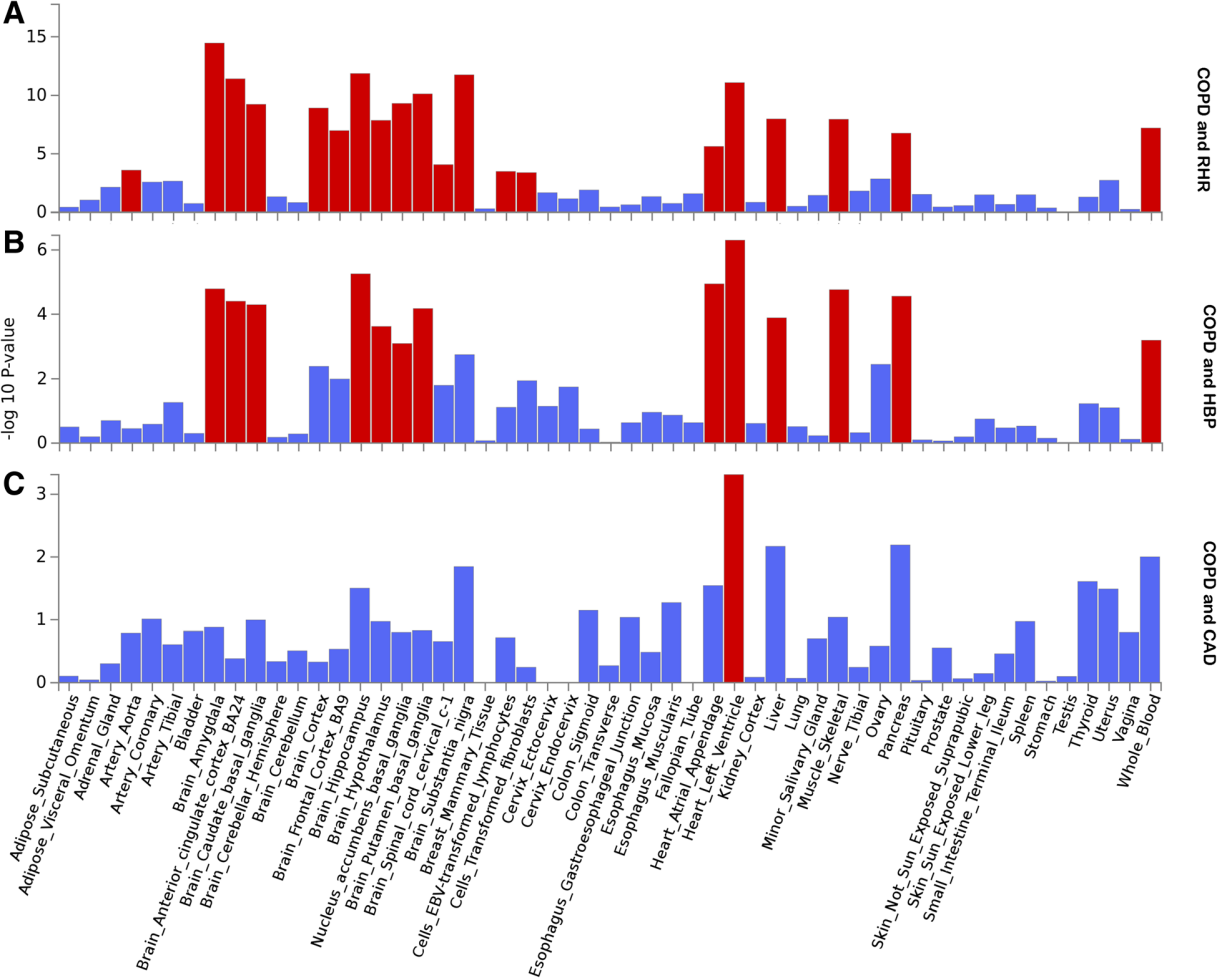
GTEx tissue enrichment analysis for expression of cross-trait-associated genes for COPD and RHR (**a**), COPD and HBP (**b**), or COPD and CAD (**c**). Red represents significant tissue enrichment after Benjamin-Hochberg correction

GTEx enrichment analysis identified 20 independent tissues that were significantly enriched (after Benjamin-Hochberg correction) for expression of cross-trait-associated genes for COPD and RHR traits, the top of which was brain amygdala (Fig. 3). In addition, all 13 independent tissues enriched for COPD and HBP trait expression overlapped with COPD and RHR traits. COPD and CAD trait expression only showed one significantly enriched tissue, heart left ventricle.

To identify associations between COPD and cardiac traits with gene expression in specific tissues, we conducted TWAS analysis in 44 GTEx tissues. A total of 231 gene–tissue pairs were significantly associated with COPD, in addition to 8504 gene–tissue pairs with RHR, 8272 gene–tissue pairs with HBP, and 805 gene–tissue pairs with CAD. Most associations were found in heart, vascular system, and lung tissues. Notably, 18 COPD-associated gene–tissue pairs were shared with RHR, 16 pairs were shared with HBP, and 2 pairs were shared with CAD (Additional file 1: Table S21).

### Effect of smoking-related genetic variants between COPD and cardiac traits

**Fig. 4:**
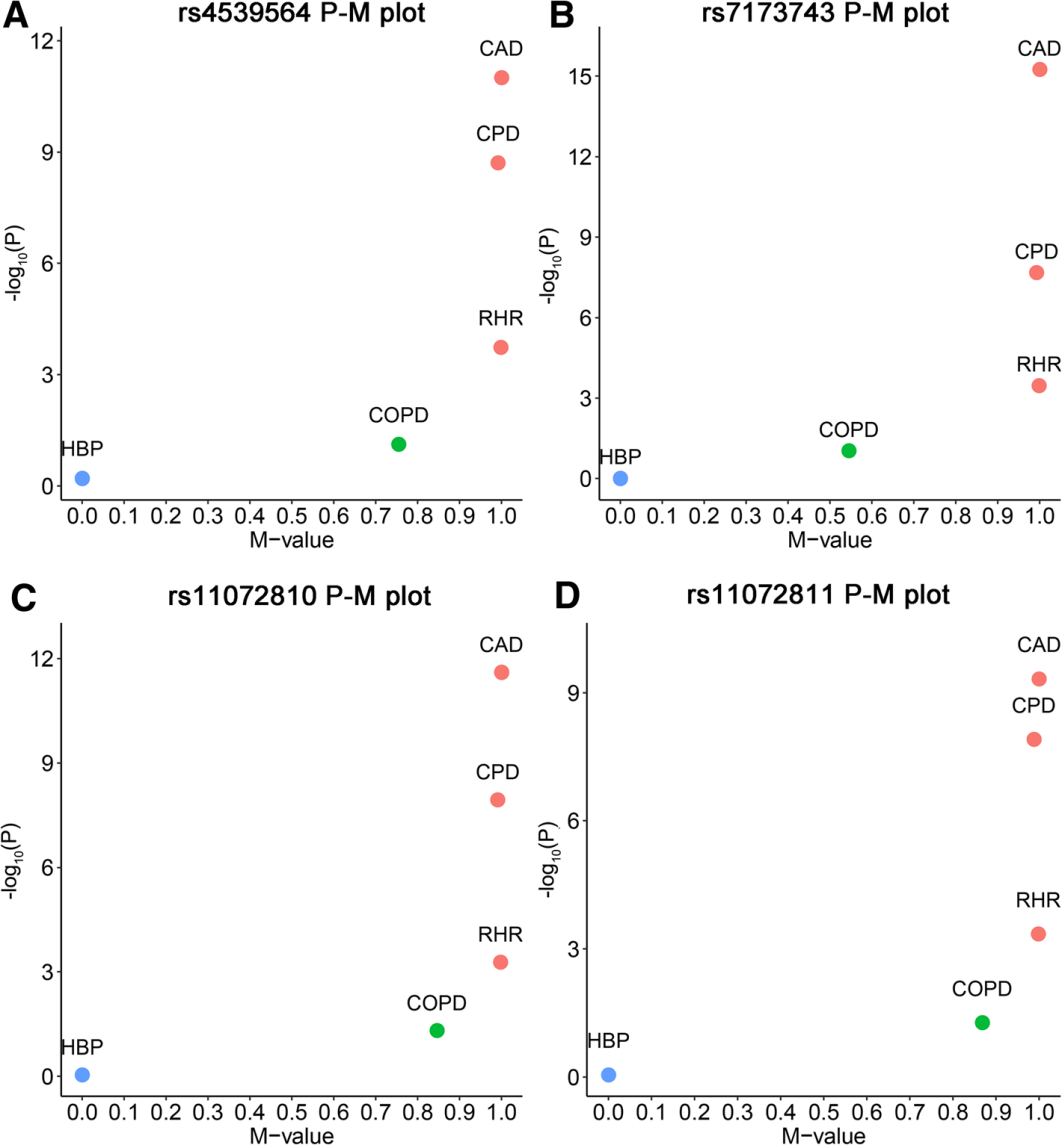
PM plots of 4 smoking related SNPs from Tobacco and Genetics Consortium that also have an effect on at least one CVD trait. **a** rs4539564, (**b**) rs7173743, (**c**) rs11072810, (**d**) rs11072811. Red dot represents the SNP has an effect on certain traits (M-value> 0.9); green dot represents the SNP may have an effect on certain traits (0.1 ≤ M-value≤0.9), but the effect is ambiguous; blue dot represents the SNP does not have an effect on certain traits (M-value< 0.1)

In the GWAS cross-trait subset effect analysis of smoking-related genetic variants, four SNPs located in the 15q25.1 region (rs4539564, rs11072810, rs11072811 and rs7173743) with CPD genetic effect, were also identified to be associated with RHR and CAD traits. These SNPs also had a moderate effect in COPD with M-values more than 0.5 (Fig. 4 and Additional file 1: Table S22).

### Causal inference

We identified a significant positive causal effect of COPD on RHR (causal estimate = 0.1374, *P* = 0.008), but not on HBP (causal estimate = 0.007, *P* = 0.35) or CAD (causal estimate = 0.004, *P* = 0.40) (Additional file 1: Table S23).

## Discussion

To our knowledge, this study is the first large-scale genome-wide analysis to investigate genetic overlap between COPD and cardiac traits. We found significant positive genome-wide genetic correlation of COPD with RHR or HBP, and a positive correlation between COPD and CAD, although this latter association failed to reach statistical significance. In the analysis of functional partitioned LDSC, we observed positive genetic correlations between COPD and cardiac traits in most annotated regions of the genome. Among them, introns, h3k9ac, and super enhancers had the highest magnitude and significance.

GWAS most frequently detects non-coding variants, and variants affecting gene expression have been shown to have pervasive effects on most diseases [46]. Histone markers like h3k9ac and h3k4me3 are some of the most essential modification markers involved in arterial pressure [47] and development of bronchial epithelial cells influencing COPD [48]. Super enhancer regions have multiple enhancers that drive transcription of genes involved in cell identity in diseases and heart development [49]. In local genetic correlation analysis, we identified multiple novel regions that have strong local genetic correlation between COPD and cardiac traits, such as the 4q31 region shared by COPD and RHR, and 11q22 and 5q32 regions shared by COPD and HBP. The 4q31 region was previously reported to have an independent association with COPD and RHR [20, 50], although it has not been identified as a shared region. By contrast, we did not observe any significant local genetic correlation between COPD and CAD.

We also discovered 21 shared loci between COPD and RHR, 22 shared loci between COPD and HBP, and 3 shared loci between COPD and CAD using cross-trait meta-analysis. Among them, we highlight the novel association of *HHIP*, *EEFSEC*, *RIN3*, *SIX5,* and *DMPK* with COPD and cardiac traits due to their potentially interesting functions.

First, the top sentinel variant for both COPD/RHR and COPD/HBP was rs7655625 near *HHIP*, known to be associated with COPD susceptibility by influencing crucial lung development signaling pathway [51]. *HHIP* is also downregulated during angiogenesis and under oxidative stress [52], and its knockdown in late endothelial progenitor cells improves endothelial angiogenesis, promoting vascular repair [53]. Another top association common to the COPD/RHR and COPD/HBP meta-analysis was with variants near *EEFSEC*, however the two analyses identified different sentinel variants. *EEFSEC* encodes a translation factor necessary for incorporation of selenocysteine into proteins associated with COPD [11] and cardiovascular events [41]. *DMPK* encodes a myotonic dystrophy protein kinase that is involved in heart cells, and *SIX5*, encodes a homeodomain-containing transcription factor that appears to function in the regulation of organogenesis [44]. Fine-mapping analysis identified multiple missense variants. For example, in meta-analysis of COPD and RHR only, we identified *RIN3* as a significant locus. Fine-mapping analysis found that rs117068593 is a missense variant in which the effect allele T results in mutation *R279C* in *RIN3*. Also, several missense variants were found in *SIX5* and *DMPK*, which are associated with COPD and CAD. However, we stress that the causal genes in these and other associated regions cannot be determined without further study.

Post-GWAS functional analyses provided biological insights to the shared genes between COPD and cardiac traits. GTEx tissue enrichment analysis identified shared genes that were significantly enriched in several tissues, including cardiovascular, nervous, and immune systems. Our findings of cardiovascular system genetic enrichment could eventually have therapeutic implications for managing COPD patients through exploration of shared mechanisms in genes such as *HHIP* [53].

Although the association between COPD/CVD and the nervous system may initially seem counterintuitive, further exploring their genetic link may provide functional and molecular understanding of their etiologies. Impaired brain function is a complication of COPD and CVD [54], which can be due to systemic inflammation, induced stress, and neurochemical abnormalities [55]. Further, stimulation of nicotinic cholinergic receptors releases a variety of neurotransmitters in the brain, which have adverse effects [55]. Nicotine-related functions in both diseases were also highlighted in our biological pathway analysis.

In TWAS analysis, we integrated data from GWAS and GTEx tissue expression to identify shared mechanistic hypotheses between COPD and cardiac traits on a tissue–gene pair level. We found 231 unique gene–tissue pairs with transcriptome-wide significant associations with COPD, in addition to 8504 with RHR, 8272 with HBP, and 805 with CAD. Most were associated with heart, vascular system, and lung tissues. Notably, 18 COPD-associated gene–tissue pairs were shared with RHR, 16 pairs were shared with HBP, and 2 pairs were shared with CAD, thus implicating specific shared regulatory features for functional follow-up.

In addition to genetic contributions to COPD and CVD, environmental, behavioral, and clinical factors also play important roles in their comorbidity. Notably, smoking is a major common environmental risk factor for both COPD and CVD. One possible mechanism linking COPD and CVD is systemic inflammation due to smoking [9]. Thus the impact of controlling such modifiable risk factor can be large. Several interventions, such as smoking cessation, exercise, drug use (e.g., statins), increased awareness of the connection between COPD and CVD, and improved collaboration between pulmonary and cardiovascular clinicians, have been shown to improve COPD and CVD and currently represent the most hopeful approaches to disease prevention and treatment [56]. While we adjusted for cigarette smoking in our ICGC COPD GWAS, other GWAS did not, and accurate measurement of exposure is challenging. Some loci such as 15q25.1 are clearly related to cigarette smoking, which is also a risk factor for CVD. Previous studies have suggested that the 15q25.1 region played a role in nicotine, alcohol, and cocaine dependence [57]. This region has been reported related to multiple diseases, such as COPD [11]. In our cross-trait subset effect analysis, we also found 4 variants in 15q25.1 region have an effect with RHR and CAD. However, interestingly, these variants were not related to COPD, suggesting that the genetic effect of cigarette smoking between COPD and CVD is complex, and not necessarily based on the same genetic variants in 15q25.1 region.

Finally, our MR analysis suggested a significant positive causal effect of COPD on RHR. One possible causal pathway example is genetic variation leading to COPD could exacerbate right ventricular diastolic dysfunction and alterations in heart rate [8]. However, our MR results should be taken with caution as other potential confounders may bias the causal relationship. For example, COPD is also known to be associated with cardiovascular autonomic neuropathy resulting in decreased parasympathetic and increased sympathetic activity, which can alter the heart rate [58]. In addition, medication use (bronchodilators) or stimulants (such as cigarettes and caffeine) may also contribute to elevated RHR in COPD patients [7].

We also acknowledge other potential limitations in this study. First, additional GWAS cohorts are not available to replicate our findings. However, we used the largest datasets available at the time of our study to perform our analyses. Genome-wide genetic correlation results were relatively weak, and did not reach significance level after multiple testing correction. However, we found a strong local genetic correlation between COPD and RHR at 4q31, between COPD and HBP at 11q22 and 5q32 regions after multiple testing correction, which highlights the genetic overlap between COPD and CVD at regional level. In addition, we identified a credible-set of SNPs that contains potential causal variants. Further functional experiments are needed to investigate the causal variants or genes. Finally, the current study was limited to assessing shared genetic factors between COPD and CVD. Future studies on shared environmental factors between COPD and CVD are needed.

## Conclusions

Understanding the genetic overlap between COPD and CVD is important for disease prevention, timely diagnosis and treatment of both diseases. Our study shows evidence of significant positive genetic correlations between COPD and cardiac traits. Shared genetic variants were fine-mapped to improve resolution and identify potential shared causal variants with exonic missense polymorphisms. We also found multiple common biological pathways and tissue enrichments, such as nicotine response, cardiovascular, brain, and immune-related tissues, which can further our understanding of the connection between these diseases. Such shared genes and pathways might serve as common drug targets in both COPD and CVD.

## Additional files

Additional file 1: **Table S1.** Summary of GWAS data. **Table S2.** SNP based heritability and genomic inflation factor estimated by LDSC. **Table S3.** Evaluation of genetic correlation between COPD and CVD related metabolic traits. **Table S4.** Partitioned genetic correlation between COPD and 3 cardiac traits. **Table S5.** Local genetic covariance analysis between COPD and RHR (only *P* < 0.01 shown in this table). **Table S6.** Local genetic coveriance analysis between COPD and HBP (only *P* < 0.01 shown in this table). **Table S7.** Local genetic covariance analysis between COPD and CAD. **Table S8.** Genome-wide significant loci by cross-trait meta-analysis at sentinel SNPs. **Table S9.** Genome-wide significant loci by cross-trait meta-analysis at sentinel SNPs. **Table S10.** Genome-wide significant loci by cross-trait meta-analysis at sentinel SNPs. **Table S11.** Detailed annotation of cross-trait meta-analysis genome-wide significant SNPs. **Table S12.** Fine-mapping credible set analysis for 21 top loci. **Table S13.** Fine-mapping credible set analysis for 22 top loci. **Table S14.** Fine-mapping credible set analysis for 3 top loci. **Table S15.** Missense variants in 99% credible set. **Table S16.** Missense variants in 99% credible-set. **Table S17.** Missense variantsin 99% credible-set. **Table S18.** GO biological process pathway analysis for COPD and RHR. **Table S19.** GO biological process pathway analysis for COPD and HBP. **Table S20.** GO biological process pathway analysis for COPD and CAD. **Table S21.** Significant overlap transcriptome-wide association analysis results. **Table S22.** Characterization of trait-specific association for the smoking related. **Table S23.** Mendelian randomization analysis between COPD and cardiac traits. (XLSX 240 kb)

Additional file 2: Online Data Supplemental Text. (DOCX 129 kb)

Additional file 3: **Figure S1.** QQ plot of resting heart rate. **Figure S2.** QQ plot of high blood pressure. **Figure S3.** Genetic Correlation between COPD and Cardiac Traits by Functional Category. (PDF 360 kb)

## Abbreviations

ASSET: Association analysis based on SubSETs
CARDIoGRAMplusC4D: Coronary ARtery DIsease Genome wide Replication and Meta-analysis (CARDIoGRAM) plus The Coronary Artery Disease (C4D) consortium
COPD: Chronic Obstructive Pulmonary Disease
CPASSOC: Cross-Phenotype Association
DIAGRAM: DIAbetes Genetics Replication And Meta-analysis consortium
ENGAGE: European Network for Genetic and Genomic Epidemiology consortium
FDR: False Discovery Rate
GIANT: The Genetic Investigation of ANthropometric Traits (GIANT) consortium
GO: Gene Ontology
GOLD: The Global Initiative for Chronic Obstructive Lung Disease
GTEx: The Genotype-Tissue Expression
HESS: Heritability Estimation from Summary Statistics
ICGC: International COPD Genomic Consortium
ISGC: International Stroke Genetics Consortium
LDSC: LD Score Regression
MR-PRESSO: Mendelian Randomization Pleiotropy RESidual Sum and Outlier
M-value: Posterior probability to evaluate if the genetic variant effect exists among traits
TAG: Tobacco and Genetics Consortium.
TWAS: Transcriptome-Wide Association Study
UKBB: UK Biobank
ρ-HESS: A tool to quantify the correlation between pairs of traits due to genetic variation at a small region in the genome
