# Supplementary material for: Genetic overlap of chronic obstructive pulmonary disease and cardiovascular disease-related traits: a large-scale genome-wide cross-trait analysis
Source: Respir Res. 2019 Apr 2;20:64. doi: 10.1186/s12931-019-1036-8 (PMC6444755; doi:10.1186/s12931-019-1036-8)
Supplement: Supplementary file 2 — Online Data Supplemental Text. (DOCX 129 kb) [file 12931_2019_1036_MOESM2_ESM.docx]

**Online Data Supplemental Text**

**Genetic overlap of chronic obstructive pulmonary disease and cardiovascular disease-related traits: A large-scale genome-wide cross-trait analysis**

Zhaozhong Zhu, Sc.D., Xiaofang Wang, M.D., Ph.D., Xihao Li, Ph.D., Yifei Lin, M.D., Ph.D., Sipeng, Shen, Cong-Lin Liu, M.D., Ph.D., Brain D. Hobbs, Kohei Hasegawa, M.D., M.P.H., Liming Liang, Ph.D., International COPD Genetics Consortium, Marike Boezen, M.D., Carlos A. Camargo, Jr., M.D., Dr.P.H., Michael H. Cho, M.D., David C. Christiani M.D.

**International COPD Genetics Consortium (ICGC) dataset**

The ICGC dataset contains 26 cohorts from worldwide populations. Four cohorts (EOCOPD, ICGN, TCGS-Poland, and TCGS-Korea) that do not have genome-wide array genotyping were excluded from this meta-analysis. Thus, the mixed population ICGC meta-analysis dataset contained 12,550 COPD cases and 46,368 controls from 22 cohorts. The European-only meta-analysis dataset excluded 5 cohorts (CHS AA, COPDGene AA, KARE, MESA AA, and MESA Hispanic) due to non-European ancestry. Detailed original descriptions can be found in a previous study([1](#_ENREF_1)). As most cohorts did not have post-bronchodilator spirometry data, we used modified definitions of GOLD criteria based on pre-bronchodilator spirometry data: FEV1 of <80% and FEV1 to forced vital capacity (FVC) ratio of <0.7 for cases; or FEV1 of >80% and FEV1/FVC of >0.7 for controls.

**UK Biobank dataset**

The UK Biobank study is a prospective study of >500,000 participants living in the UK. In total, 503,325 participants who registered in the National Health Service with ages ranging 40–69 years were recruited out of 9.2 million mailed invitations. Baseline data were collected using questionnaires, and anthropometric assessments were performed. All detailed genotyping, quality con­trol, and imputation procedures are described at the UK Biobank website (http://biobank.ctsu.ox.ac.uk). Out of 500,000 subjects in the UK Biobank, currently ~153,000 individuals have been genotyped for ~800,000 SNPs using Affymetrix facilities. Population structure was captured by principal component analysis on ~500,000 UK Biobank samples using ~100,000 SNPs. Quality control filters were applied before phasing. Data were prephased using SHAPEIT3([2](#_ENREF_2)). Phase 3 1000 Genomes Project and UK10k data was used as a reference panel for imputation. Imputation was carried out using the IMPUTE3 program, which uses the same algorithm as IMPUTE2([3](#_ENREF_3)). In total, 92,457,989 variants were imputed for all individuals. QCTOOL (<http://www.well.ox.ac.uk/~gav/qctool>) was used to calculate minor allele frequency and imputation information score of each imputed variant. To avoid confounding effects, we excluded data from non-white individuals for this analysis.

We used two cardiac traits from this cohort: high blood pressure (HBP) (N = 458,554) and resting heart rate (RHR) (N = 458,969). We used data field 6150 from the participant questionnaire to determine the doctor-diagnosed HBP phenotype. This data field contains the question: “Has a doctor ever told you that you have had any of the following conditions?” Participants could select more than one answer from the following: heart attack; angina; stroke; high blood pressure; none of the above; and prefer not to answer. If participants chose either “none of the above” or “prefer not to answer”, they could not select other answers. Among vascular/heart problems, we focused on HBP due to its largest sample size (N_case_ = 144,793) compared to other phenotypes to ensure highly powered and non-biased association results([4](#_ENREF_4)). We used both data field 102 and 95 for RHR. RHR was assessed via two methods: automated reading during blood pressure measurement (in 501,340 participants); and pulse waveform obtained from the finger with an infrared sensor during arterial stiffness measurement (in 193,472 participants). RHR was averaged if multiple measurements were available for one individual([5](#_ENREF_5)). We used data field 4079 for diastolic blood pressure and 4080 for systolic blood pressure. The blood pressure measurements was based on automated reading. The details of blood pressure measurement protocol can be found through UK Biobank website (URL： https://biobank.ctsu.ox.ac.uk/crystal/docs/Bloodpressure.pdf.)

All participants from this study provided UK Biobank-acquired informed consent and provided data according to the UK Biobank protocol. We have complied with all ethical regulations according to UK Biobank policy. This research was approved and conducted using the UK Biobank under application number 16549.

**Cardiovascular disease (CVD) related metabolic traits GWAS datasets**

We retrieved summary statistics for CVD related metabolic traits from publically available GWAS studies, including body mass index (BMI) ([6](#_ENREF_6)) (N= 236,231 ) and waist-to-hip ratio (WHR) ([7](#_ENREF_7)) (N= 142,762 ) from the GIANT Consortium, T2D from the DIAGRAM Consortium ([8](#_ENREF_8)) (N= 159,208 ), and blood lipids (HDL-C [N= 60,812], LDL-C [N= 58,381], TC [N= 60,027], and TG [N= 62,166]) from ENGAGE Consortium ([9](#_ENREF_9)). Details of each of the datasets can be found in Table E1.

**GWAS analysis of UK Biobank data**

To account for relatedness, the association between cardiac traits in UK Biobank data and imputed SNPs was carried out using BOLT-linear mixed model (LMM)([4](#_ENREF_4)). The output of BOLT-LMM linear regression was transformed into log odds ratio (logOR) for HBP binary phenotype using the following equation:

$$logOR=\frac{Beta_{BOLT-LMM}}{\frac{N_{case}}{N_{control}}*\left( 1-\frac{N_{case}}{N_{control}} \right)}$$

Association analysis was adjusted for age, sex, smoking, genotyping array, and 20 ancestry principal components.

**Linkage disequilibrium (LD) score regression (LDSC) analysis**

We conducted post-GWAS genetic correlation analysis with LDSC using all SNPs after merging with HapMap3 SNP, excluding the HLA region. LDSC estimates genetic correlations from summary statistics using the fact that GWAS effect size estimate for each SNP incorporates the effects of all SNPs in LD with that SNP. SNPs in a high LD region would have higher χ2 statistics than SNPs in a low LD region, and a similar relationship is observed when single-study test statistics are replaced with the product of z-scores from two studies of traits with some correlation([10](#_ENREF_10)). LDSC analysis applied a self-estimated intercept to account for shared subjects between studies([11](#_ENREF_11)). European-ancestry subjects were used in LDSC analysis for each trait if available.

**Association analysis based on subsets (ASSET)**

ASSET is a generalized fixed-effects meta-analysis model that combines effect estimate and standard error of GWAS of related but distinct traits to identify promising directions to discover loci with small but common pleiotropic effects. The ASSET method explores subsets of studies for the presence of true association signals that are either in the same direction or opposite directions([12](#_ENREF_12)). When *S* represents a set of study traits selected from *K* studies, meta-analysis statistics of the one-sided test ASSET is defined as:

$$Z_{max-ASSET}={max}_{S\in\boldsymbol{S}} | Z\left( S \right)| ={max}_{S\in\boldsymbol{S}}|\sum_{k\in S} \sqrt{\pi_{k}\left( S \right)}Z_{k}|$$

where *S* is all possible 2*^K^*–1 subsets of K studies, and $\pi_{k}\left( T \right)=n_{k}/\sum_{k\in T} n_{k}$ represents sample size of the study *K* relative to total sample size of the given subset *S*.

An advantage of using ASSET is that it can account for correlation among studies/subjects that might arise due to shared subjects across distinct studies or due to correlation among related traits in the same study by using case–control overlap matrices. If Z(A) and Z(B) denote Z statistics for the association test for a SNP from case–control studies A and B with an arbitrary amount of overlap between subjects, then—under the null hypothesis of no association and the assumption that there is no covariate adjustment—the correlation between statistics is given by

$$Corr\left\{ Z\left( A \right),Z(B) \right\}=\sqrt{\frac{n_{A}^{(1)}n_{A}^{(0)}}{N_{A}}}\sqrt{\frac{n_{B}^{(1)}n_{B}^{(0)}}{N_{B}}}\left[ \frac{n_{AB}^{(11)}}{n_{A}^{(1)}n_{B}^{(1)}}-\frac{n_{AB}^{\left( 10 \right)}}{n_{A}^{\left( 1 \right)}n_{B}^{\left( 0 \right)}}-\frac{n_{AB}^{\left( 01 \right)}}{n_{A}^{\left( 0 \right)}n_{B}^{\left( 1 \right)}}+\frac{n_{AB}^{(00)}}{n_{A}^{(0)}n_{B}^{(0)}} \right]$$

where $n_{A}^{(1)}$, $n_{A}^{(0)}$, and *N_A_* are the number of cases, controls, and subjects, respectively, in study A; $n_{B}^{(1)}$, $n_{B}^{(0)}$, and *N_B_* are the number of cases, controls, and subjects, respectively, in study B; and $n_{AB}^{(ij)}$ represents the number of subjects with different phenotype categories (i,j) ∈ (0,1) that overlap between studies A and B. For example, $n_{AB}^{(11)}$ denotes the number of shared cases between studies A and B, which is 6,177 in our study; $n_{AB}^{(10)}$ denotes the number of individuals who are treated as cases in study A but as controls in study B, which is 0 in our study; $n_{AB}^{(01)}$ denotes the number of individuals who are treated as controls in study A but as cases in study B, which is 0 in our study; and $n_{AB}^{(00)}$ denotes the number of shared controls between studies A and B, which is 76,768 in our study([12](#_ENREF_12)).

**Cross-phenotype association (CPASSOC)**

CPASSOC combines effect estimates and standard errors of GWAS summary statistics to test association hypotheses of the SNP with both traits. Similar to ASSET, CPASSOC assumes that effects may exist only within a subset of study traits([13](#_ENREF_13)). However, unlike those two methods, CPASSOC identifies a subset of studies with effects by sequentially adding a trait by an incremental order of association significance. Among sequentially examined subsets, the highest meta-statistic is selected. A heterogonous version of CPASSOC (SHet) was used in this study.

SHet is a cross-phenotype meta-analysis method based on the fixed effect model. It can be viewed as maximum of the weighted sum of trait-specific test statistics satisfying different thresholds, which is closely related to a gamma distribution. It is more powerful when there is heterogonous effects between studies, which is common in meta-analysis of different phenotypes([13](#_ENREF_13), [14](#_ENREF_14)). SHet uses the sample size for a trait as a weight instead of a variance. It can also account for correlation due to overlapping or related subjects within and among different studies or cohorts. We used the following equation for SHet:

$$S\left( \tau\right)=\frac{e^{\tau}\left( R\left( \tau\right)W\left( \tau\right) \right)^{-1}U\left( \tau\right)\left( e^{T}\left( R\left( \tau\right)W\left( \tau\right) \right)^{-1}U\left( \tau\right) \right)^{T}}{e^{T}W\left( \tau\right)^{-1}R\left( \tau\right)^{-1}W\left( \tau\right)^{-1}e}$$

$$S_{Het}=\max_{\tau>0} S(\tau)$$

where $R\left( \tau\right)$ is a submatrix of *R* representing the correlation matrix between study traits, $U\left( \tau\right)$ is the sub-vector of the Wald test statistic $U$ satisfying $U_{jk}>\tau$, and $W\left( \tau\right)$ is the diagonal submatrix of *W* corresponding to $T\left( \tau\right)$.

**References**

1. Hobbs BD, de Jong K, Lamontagne M, Bosse Y, Shrine N, Artigas MS, Wain LV, Hall IP, Jackson VE, Wyss AB, London SJ, North KE, Franceschini N, Strachan DP, Beaty TH, Hokanson JE, Crapo JD, Castaldi PJ, Chase RP, Bartz TM, Heckbert SR, Psaty BM, Gharib SA, Zanen P, Lammers JW, Oudkerk M, Groen HJ, Locantore N, Tal-Singer R, Rennard SI, Vestbo J, Timens W, Pare PD, Latourelle JC, Dupuis J, O'Connor GT, Wilk JB, Kim WJ, Lee MK, Oh YM, Vonk JM, de Koning HJ, Leng S, Belinsky SA, Tesfaigzi Y, Manichaikul A, Wang XQ, Rich SS, Barr RG, Sparrow D, Litonjua AA, Bakke P, Gulsvik A, Lahousse L, Brusselle GG, Stricker BH, Uitterlinden AG, Ampleford EJ, Bleecker ER, Woodruff PG, Meyers DA, Qiao D, Lomas DA, Yim JJ, Kim DK, Hawrylkiewicz I, Sliwinski P, Hardin M, Fingerlin TE, Schwartz DA, Postma DS, MacNee W, Tobin MD, Silverman EK, Boezen HM, Cho MH, Investigators CO, Investigators E, LifeLines I, Group SR, International CGNI, Investigators UKB, International CGC. Genetic loci associated with chronic obstructive pulmonary disease overlap with loci for lung function and pulmonary fibrosis. *Nat Genet* 2017; 49: 426-432.

2. O'Connell J, Sharp K, Shrine N, Wain L, Hall I, Tobin M, Zagury JF, Delaneau O, Marchini J. Haplotype estimation for biobank-scale data sets. *Nature genetics* 2016; 48: 817-820.

3. Howie BN, Donnelly P, Marchini J. A flexible and accurate genotype imputation method for the next generation of genome-wide association studies. *PLoS genetics* 2009; 5: e1000529.

4. Loh PR, Tucker G, Bulik-Sullivan BK, Vilhjalmsson BJ, Finucane HK, Salem RM, Chasman DI, Ridker PM, Neale BM, Berger B, Patterson N, Price AL. Efficient Bayesian mixed-model analysis increases association power in large cohorts. *Nature genetics* 2015; 47: 284-290.

5. Eppinga RN, Hagemeijer Y, Burgess S, Hinds DA, Stefansson K, Gudbjartsson DF, van Veldhuisen DJ, Munroe PB, Verweij N, van der Harst P. Identification of genomic loci associated with resting heart rate and shared genetic predictors with all-cause mortality. *Nature genetics* 2016; 48: 1557-1563.

6. Locke AE, Kahali B, Berndt SI, Justice AE, Pers TH, Day FR, Powell C, Vedantam S, Buchkovich ML, Yang J, Croteau-Chonka DC, Esko T, Fall T, Ferreira T, Gustafsson S, Kutalik Z, Luan J, Magi R, Randall JC, Winkler TW, Wood AR, Workalemahu T, Faul JD, Smith JA, Zhao JH, Zhao W, Chen J, Fehrmann R, Hedman AK, Karjalainen J, Schmidt EM, Absher D, Amin N, Anderson D, Beekman M, Bolton JL, Bragg-Gresham JL, Buyske S, Demirkan A, Deng G, Ehret GB, Feenstra B, Feitosa MF, Fischer K, Goel A, Gong J, Jackson AU, Kanoni S, Kleber ME, Kristiansson K, Lim U, Lotay V, Mangino M, Leach IM, Medina-Gomez C, Medland SE, Nalls MA, Palmer CD, Pasko D, Pechlivanis S, Peters MJ, Prokopenko I, Shungin D, Stancakova A, Strawbridge RJ, Sung YJ, Tanaka T, Teumer A, Trompet S, van der Laan SW, van Setten J, Van Vliet-Ostaptchouk JV, Wang Z, Yengo L, Zhang W, Isaacs A, Albrecht E, Arnlov J, Arscott GM, Attwood AP, Bandinelli S, Barrett A, Bas IN, Bellis C, Bennett AJ, Berne C, Blagieva R, Bluher M, Bohringer S, Bonnycastle LL, Bottcher Y, Boyd HA, Bruinenberg M, Caspersen IH, Chen YI, Clarke R, Daw EW, de Craen AJM, Delgado G, Dimitriou M, Doney ASF, Eklund N, Estrada K, Eury E, Folkersen L, Fraser RM, Garcia ME, Geller F, Giedraitis V, Gigante B, Go AS, Golay A, Goodall AH, Gordon SD, Gorski M, Grabe HJ, Grallert H, Grammer TB, Grassler J, Gronberg H, Groves CJ, Gusto G, Haessler J, Hall P, Haller T, Hallmans G, Hartman CA, Hassinen M, Hayward C, Heard-Costa NL, Helmer Q, Hengstenberg C, Holmen O, Hottenga JJ, James AL, Jeff JM, Johansson A, Jolley J, Juliusdottir T, Kinnunen L, Koenig W, Koskenvuo M, Kratzer W, Laitinen J, Lamina C, Leander K, Lee NR, Lichtner P, Lind L, Lindstrom J, Lo KS, Lobbens S, Lorbeer R, Lu Y, Mach F, Magnusson PKE, Mahajan A, McArdle WL, McLachlan S, Menni C, Merger S, Mihailov E, Milani L, Moayyeri A, Monda KL, Morken MA, Mulas A, Muller G, Muller-Nurasyid M, Musk AW, Nagaraja R, Nothen MM, Nolte IM, Pilz S, Rayner NW, Renstrom F, Rettig R, Ried JS, Ripke S, Robertson NR, Rose LM, Sanna S, Scharnagl H, Scholtens S, Schumacher FR, Scott WR, Seufferlein T, Shi J, Smith AV, Smolonska J, Stanton AV, Steinthorsdottir V, Stirrups K, Stringham HM, Sundstrom J, Swertz MA, Swift AJ, Syvanen AC, Tan ST, Tayo BO, Thorand B, Thorleifsson G, Tyrer JP, Uh HW, Vandenput L, Verhulst FC, Vermeulen SH, Verweij N, Vonk JM, Waite LL, Warren HR, Waterworth D, Weedon MN, Wilkens LR, Willenborg C, Wilsgaard T, Wojczynski MK, Wong A, Wright AF, Zhang Q, LifeLines Cohort S, Brennan EP, Choi M, Dastani Z, Drong AW, Eriksson P, Franco-Cereceda A, Gadin JR, Gharavi AG, Goddard ME, Handsaker RE, Huang J, Karpe F, Kathiresan S, Keildson S, Kiryluk K, Kubo M, Lee JY, Liang L, Lifton RP, Ma B, McCarroll SA, McKnight AJ, Min JL, Moffatt MF, Montgomery GW, Murabito JM, Nicholson G, Nyholt DR, Okada Y, Perry JRB, Dorajoo R, Reinmaa E, Salem RM, Sandholm N, Scott RA, Stolk L, Takahashi A, Tanaka T, van 't Hooft FM, Vinkhuyzen AAE, Westra HJ, Zheng W, Zondervan KT, Consortium AD, Group A-BW, Consortium CAD, Consortium CK, Glgc, Icbp, Investigators M, Mu TC, Consortium MI, Consortium P, ReproGen C, Consortium G, International Endogene C, Heath AC, Arveiler D, Bakker SJL, Beilby J, Bergman RN, Blangero J, Bovet P, Campbell H, Caulfield MJ, Cesana G, Chakravarti A, Chasman DI, Chines PS, Collins FS, Crawford DC, Cupples LA, Cusi D, Danesh J, de Faire U, den Ruijter HM, Dominiczak AF, Erbel R, Erdmann J, Eriksson JG, Farrall M, Felix SB, Ferrannini E, Ferrieres J, Ford I, Forouhi NG, Forrester T, Franco OH, Gansevoort RT, Gejman PV, Gieger C, Gottesman O, Gudnason V, Gyllensten U, Hall AS, Harris TB, Hattersley AT, Hicks AA, Hindorff LA, Hingorani AD, Hofman A, Homuth G, Hovingh GK, Humphries SE, Hunt SC, Hypponen E, Illig T, Jacobs KB, Jarvelin MR, Jockel KH, Johansen B, Jousilahti P, Jukema JW, Jula AM, Kaprio J, Kastelein JJP, Keinanen-Kiukaanniemi SM, Kiemeney LA, Knekt P, Kooner JS, Kooperberg C, Kovacs P, Kraja AT, Kumari M, Kuusisto J, Lakka TA, Langenberg C, Marchand LL, Lehtimaki T, Lyssenko V, Mannisto S, Marette A, Matise TC, McKenzie CA, McKnight B, Moll FL, Morris AD, Morris AP, Murray JC, Nelis M, Ohlsson C, Oldehinkel AJ, Ong KK, Madden PAF, Pasterkamp G, Peden JF, Peters A, Postma DS, Pramstaller PP, Price JF, Qi L, Raitakari OT, Rankinen T, Rao DC, Rice TK, Ridker PM, Rioux JD, Ritchie MD, Rudan I, Salomaa V, Samani NJ, Saramies J, Sarzynski MA, Schunkert H, Schwarz PEH, Sever P, Shuldiner AR, Sinisalo J, Stolk RP, Strauch K, Tonjes A, Tregouet DA, Tremblay A, Tremoli E, Virtamo J, Vohl MC, Volker U, Waeber G, Willemsen G, Witteman JC, Zillikens MC, Adair LS, Amouyel P, Asselbergs FW, Assimes TL, Bochud M, Boehm BO, Boerwinkle E, Bornstein SR, Bottinger EP, Bouchard C, Cauchi S, Chambers JC, Chanock SJ, Cooper RS, de Bakker PIW, Dedoussis G, Ferrucci L, Franks PW, Froguel P, Groop LC, Haiman CA, Hamsten A, Hui J, Hunter DJ, Hveem K, Kaplan RC, Kivimaki M, Kuh D, Laakso M, Liu Y, Martin NG, Marz W, Melbye M, Metspalu A, Moebus S, Munroe PB, Njolstad I, Oostra BA, Palmer CNA, Pedersen NL, Perola M, Perusse L, Peters U, Power C, Quertermous T, Rauramaa R, Rivadeneira F, Saaristo TE, Saleheen D, Sattar N, Schadt EE, Schlessinger D, Slagboom PE, Snieder H, Spector TD, Thorsteinsdottir U, Stumvoll M, Tuomilehto J, Uitterlinden AG, Uusitupa M, van der Harst P, Walker M, Wallaschofski H, Wareham NJ, Watkins H, Weir DR, Wichmann HE, Wilson JF, Zanen P, Borecki IB, Deloukas P, Fox CS, Heid IM, O'Connell JR, Strachan DP, Stefansson K, van Duijn CM, Abecasis GR, Franke L, Frayling TM, McCarthy MI, Visscher PM, Scherag A, Willer CJ, Boehnke M, Mohlke KL, Lindgren CM, Beckmann JS, Barroso I, North KE, Ingelsson E, Hirschhorn JN, Loos RJF, Speliotes EK. Genetic studies of body mass index yield new insights for obesity biology. *Nature* 2015; 518: 197-206.

7. Shungin D, Winkler TW, Croteau-Chonka DC, Ferreira T, Locke AE, Magi R, Strawbridge RJ, Pers TH, Fischer K, Justice AE, Workalemahu T, Wu JMW, Buchkovich ML, Heard-Costa NL, Roman TS, Drong AW, Song C, Gustafsson S, Day FR, Esko T, Fall T, Kutalik Z, Luan J, Randall JC, Scherag A, Vedantam S, Wood AR, Chen J, Fehrmann R, Karjalainen J, Kahali B, Liu CT, Schmidt EM, Absher D, Amin N, Anderson D, Beekman M, Bragg-Gresham JL, Buyske S, Demirkan A, Ehret GB, Feitosa MF, Goel A, Jackson AU, Johnson T, Kleber ME, Kristiansson K, Mangino M, Leach IM, Medina-Gomez C, Palmer CD, Pasko D, Pechlivanis S, Peters MJ, Prokopenko I, Stancakova A, Sung YJ, Tanaka T, Teumer A, Van Vliet-Ostaptchouk JV, Yengo L, Zhang W, Albrecht E, Arnlov J, Arscott GM, Bandinelli S, Barrett A, Bellis C, Bennett AJ, Berne C, Bluher M, Bohringer S, Bonnet F, Bottcher Y, Bruinenberg M, Carba DB, Caspersen IH, Clarke R, Daw EW, Deelen J, Deelman E, Delgado G, Doney AS, Eklund N, Erdos MR, Estrada K, Eury E, Friedrich N, Garcia ME, Giedraitis V, Gigante B, Go AS, Golay A, Grallert H, Grammer TB, Grassler J, Grewal J, Groves CJ, Haller T, Hallmans G, Hartman CA, Hassinen M, Hayward C, Heikkila K, Herzig KH, Helmer Q, Hillege HL, Holmen O, Hunt SC, Isaacs A, Ittermann T, James AL, Johansson I, Juliusdottir T, Kalafati IP, Kinnunen L, Koenig W, Kooner IK, Kratzer W, Lamina C, Leander K, Lee NR, Lichtner P, Lind L, Lindstrom J, Lobbens S, Lorentzon M, Mach F, Magnusson PK, Mahajan A, McArdle WL, Menni C, Merger S, Mihailov E, Milani L, Mills R, Moayyeri A, Monda KL, Mooijaart SP, Muhleisen TW, Mulas A, Muller G, Muller-Nurasyid M, Nagaraja R, Nalls MA, Narisu N, Glorioso N, Nolte IM, Olden M, Rayner NW, Renstrom F, Ried JS, Robertson NR, Rose LM, Sanna S, Scharnagl H, Scholtens S, Sennblad B, Seufferlein T, Sitlani CM, Smith AV, Stirrups K, Stringham HM, Sundstrom J, Swertz MA, Swift AJ, Syvanen AC, Tayo BO, Thorand B, Thorleifsson G, Tomaschitz A, Troffa C, van Oort FV, Verweij N, Vonk JM, Waite LL, Wennauer R, Wilsgaard T, Wojczynski MK, Wong A, Zhang Q, Zhao JH, Brennan EP, Choi M, Eriksson P, Folkersen L, Franco-Cereceda A, Gharavi AG, Hedman AK, Hivert MF, Huang J, Kanoni S, Karpe F, Keildson S, Kiryluk K, Liang L, Lifton RP, Ma B, McKnight AJ, McPherson R, Metspalu A, Min JL, Moffatt MF, Montgomery GW, Murabito JM, Nicholson G, Nyholt DR, Olsson C, Perry JR, Reinmaa E, Salem RM, Sandholm N, Schadt EE, Scott RA, Stolk L, Vallejo EE, Westra HJ, Zondervan KT, Consortium AD, Consortium CAD, Consortium CK, Consortium G, Consortium G, Glgc, Icbp, International Endogene C, LifeLines Cohort S, Investigators M, Mu TC, Consortium P, ReproGen C, Amouyel P, Arveiler D, Bakker SJ, Beilby J, Bergman RN, Blangero J, Brown MJ, Burnier M, Campbell H, Chakravarti A, Chines PS, Claudi-Boehm S, Collins FS, Crawford DC, Danesh J, de Faire U, de Geus EJ, Dorr M, Erbel R, Eriksson JG, Farrall M, Ferrannini E, Ferrieres J, Forouhi NG, Forrester T, Franco OH, Gansevoort RT, Gieger C, Gudnason V, Haiman CA, Harris TB, Hattersley AT, Heliovaara M, Hicks AA, Hingorani AD, Hoffmann W, Hofman A, Homuth G, Humphries SE, Hypponen E, Illig T, Jarvelin MR, Johansen B, Jousilahti P, Jula AM, Kaprio J, Kee F, Keinanen-Kiukaanniemi SM, Kooner JS, Kooperberg C, Kovacs P, Kraja AT, Kumari M, Kuulasmaa K, Kuusisto J, Lakka TA, Langenberg C, Le Marchand L, Lehtimaki T, Lyssenko V, Mannisto S, Marette A, Matise TC, McKenzie CA, McKnight B, Musk AW, Mohlenkamp S, Morris AD, Nelis M, Ohlsson C, Oldehinkel AJ, Ong KK, Palmer LJ, Penninx BW, Peters A, Pramstaller PP, Raitakari OT, Rankinen T, Rao DC, Rice TK, Ridker PM, Ritchie MD, Rudan I, Salomaa V, Samani NJ, Saramies J, Sarzynski MA, Schwarz PE, Shuldiner AR, Staessen JA, Steinthorsdottir V, Stolk RP, Strauch K, Tonjes A, Tremblay A, Tremoli E, Vohl MC, Volker U, Vollenweider P, Wilson JF, Witteman JC, Adair LS, Bochud M, Boehm BO, Bornstein SR, Bouchard C, Cauchi S, Caulfield MJ, Chambers JC, Chasman DI, Cooper RS, Dedoussis G, Ferrucci L, Froguel P, Grabe HJ, Hamsten A, Hui J, Hveem K, Jockel KH, Kivimaki M, Kuh D, Laakso M, Liu Y, Marz W, Munroe PB, Njolstad I, Oostra BA, Palmer CN, Pedersen NL, Perola M, Perusse L, Peters U, Power C, Quertermous T, Rauramaa R, Rivadeneira F, Saaristo TE, Saleheen D, Sinisalo J, Slagboom PE, Snieder H, Spector TD, Stefansson K, Stumvoll M, Tuomilehto J, Uitterlinden AG, Uusitupa M, van der Harst P, Veronesi G, Walker M, Wareham NJ, Watkins H, Wichmann HE, Abecasis GR, Assimes TL, Berndt SI, Boehnke M, Borecki IB, Deloukas P, Franke L, Frayling TM, Groop LC, Hunter DJ, Kaplan RC, O'Connell JR, Qi L, Schlessinger D, Strachan DP, Thorsteinsdottir U, van Duijn CM, Willer CJ, Visscher PM, Yang J, Hirschhorn JN, Zillikens MC, McCarthy MI, Speliotes EK, North KE, Fox CS, Barroso I, Franks PW, Ingelsson E, Heid IM, Loos RJ, Cupples LA, Morris AP, Lindgren CM, Mohlke KL. New genetic loci link adipose and insulin biology to body fat distribution. *Nature* 2015; 518: 187-196.

8. Scott RA, Scott LJ, Magi R, Marullo L, Gaulton KJ, Kaakinen M, Pervjakova N, Pers TH, Johnson AD, Eicher JD, Jackson AU, Ferreira T, Lee Y, Ma C, Steinthorsdottir V, Thorleifsson G, Qi L, Van Zuydam NR, Mahajan A, Chen H, Almgren P, Voight BF, Grallert H, Muller-Nurasyid M, Ried JS, Rayner NW, Robertson N, Karssen LC, van Leeuwen EM, Willems SM, Fuchsberger C, Kwan P, Teslovich TM, Chanda P, Li M, Lu Y, Dina C, Thuillier D, Yengo L, Jiang L, Sparso T, Kestler HA, Chheda H, Eisele L, Gustafsson S, Franberg M, Strawbridge RJ, Benediktsson R, Hreidarsson AB, Kong A, Sigurethsson G, Kerrison ND, Luan J, Liang L, Meitinger T, Roden M, Thorand B, Esko T, Mihailov E, Fox C, Liu CT, Rybin D, Isomaa B, Lyssenko V, Tuomi T, Couper DJ, Pankow JS, Grarup N, Have CT, Jorgensen ME, Jorgensen T, Linneberg A, Cornelis MC, van Dam RM, Hunter DJ, Kraft P, Sun Q, Edkins S, Owen KR, Perry JRB, Wood AR, Zeggini E, Tajes-Fernandes J, Abecasis GR, Bonnycastle LL, Chines PS, Stringham HM, Koistinen HA, Kinnunen L, Sennblad B, Muhleisen TW, Nothen MM, Pechlivanis S, Baldassarre D, Gertow K, Humphries SE, Tremoli E, Klopp N, Meyer J, Steinbach G, Wennauer R, Eriksson JG, Mnnisto S, Peltonen L, Tikkanen E, Charpentier G, Eury E, Lobbens S, Gigante B, Leander K, McLeod O, Bottinger EP, Gottesman O, Ruderfer D, Bluher M, Kovacs P, Tonjes A, Maruthur NM, Scapoli C, Erbel R, Jockel KH, Moebus S, de Faire U, Hamsten A, Stumvoll M, Deloukas P, Donnelly PJ, Frayling TM, Hattersley AT, Ripatti S, Salomaa V, Pedersen NL, Boehm BO, Bergman RN, Collins FS, Mohlke KL, Tuomilehto J, Hansen T, Pedersen O, Barroso I, Lannfelt L, Ingelsson E, Lind L, Lindgren CM, Cauchi S, Froguel P, Loos RJF, Balkau B, Boeing H, Franks PW, Barricarte Gurrea A, Palli D, van der Schouw YT, Altshuler D, Groop LC, Langenberg C, Wareham NJ, Sijbrands E, van Duijn CM, Florez JC, Meigs JB, Boerwinkle E, Gieger C, Strauch K, Metspalu A, Morris AD, Palmer CNA, Hu FB, Thorsteinsdottir U, Stefansson K, Dupuis J, Morris AP, Boehnke M, McCarthy MI, Prokopenko I, Replication DIG, Meta-analysis C. An Expanded Genome-Wide Association Study of Type 2 Diabetes in Europeans. *Diabetes* 2017; 66: 2888-2902.

9. Surakka I, Horikoshi M, Magi R, Sarin AP, Mahajan A, Lagou V, Marullo L, Ferreira T, Miraglio B, Timonen S, Kettunen J, Pirinen M, Karjalainen J, Thorleifsson G, Hagg S, Hottenga JJ, Isaacs A, Ladenvall C, Beekman M, Esko T, Ried JS, Nelson CP, Willenborg C, Gustafsson S, Westra HJ, Blades M, de Craen AJ, de Geus EJ, Deelen J, Grallert H, Hamsten A, Havulinna AS, Hengstenberg C, Houwing-Duistermaat JJ, Hypponen E, Karssen LC, Lehtimaki T, Lyssenko V, Magnusson PK, Mihailov E, Muller-Nurasyid M, Mpindi JP, Pedersen NL, Penninx BW, Perola M, Pers TH, Peters A, Rung J, Smit JH, Steinthorsdottir V, Tobin MD, Tsernikova N, van Leeuwen EM, Viikari JS, Willems SM, Willemsen G, Schunkert H, Erdmann J, Samani NJ, Kaprio J, Lind L, Gieger C, Metspalu A, Slagboom PE, Groop L, van Duijn CM, Eriksson JG, Jula A, Salomaa V, Boomsma DI, Power C, Raitakari OT, Ingelsson E, Jarvelin MR, Thorsteinsdottir U, Franke L, Ikonen E, Kallioniemi O, Pietiainen V, Lindgren CM, Stefansson K, Palotie A, McCarthy MI, Morris AP, Prokopenko I, Ripatti S, Consortium E. The impact of low-frequency and rare variants on lipid levels. *Nat Genet* 2015; 47: 589-597.

10. Bulik-Sullivan BK, Loh PR, Finucane HK, Ripke S, Yang J, Schizophrenia Working Group of the Psychiatric Genomics C, Patterson N, Daly MJ, Price AL, Neale BM. LD Score regression distinguishes confounding from polygenicity in genome-wide association studies. *Nat Genet* 2015; 47: 291-295.

11. Bulik-Sullivan B, Finucane HK, Anttila V, Gusev A, Day FR, Loh PR, ReproGen C, Psychiatric Genomics C, Genetic Consortium for Anorexia Nervosa of the Wellcome Trust Case Control C, Duncan L, Perry JR, Patterson N, Robinson EB, Daly MJ, Price AL, Neale BM. An atlas of genetic correlations across human diseases and traits. *Nat Genet* 2015; 47: 1236-1241.

12. Bhattacharjee S, Rajaraman P, Jacobs KB, Wheeler WA, Melin BS, Hartge P, GliomaScan C, Yeager M, Chung CC, Chanock SJ, Chatterjee N. A subset-based approach improves power and interpretation for the combined analysis of genetic association studies of heterogeneous traits. *American journal of human genetics* 2012; 90: 821-835.

13. Zhu X, Feng T, Tayo BO, Liang J, Young JH, Franceschini N, Smith JA, Yanek LR, Sun YV, Edwards TL, Chen W, Nalls M, Fox E, Sale M, Bottinger E, Rotimi C, Consortium CB, Liu Y, McKnight B, Liu K, Arnett DK, Chakravati A, Cooper RS, Redline S. Meta-analysis of correlated traits via summary statistics from GWASs with an application in hypertension. *Am J Hum Genet* 2015; 96: 21-36.

14. Zhu Z, Anttila V, Smoller JW, Lee PH. Statistical power and utility of meta-analysis methods for cross-phenotype genome-wide association studies. *PloS one* 2018; 13: e0193256.

15. Verbanck M, Chen CY, Neale B, Do R. Detection of widespread horizontal pleiotropy in causal relationships inferred from Mendelian randomization between complex traits and diseases. *Nature genetics* 2018; 50: 693-698.
