# Supplementary material for: Genetic overlap of chronic obstructive pulmonary disease and cardiovascular disease-related traits: a large-scale genome-wide cross-trait analysis
Source: Respir Res. 2019 Apr 2;20:64. doi: 10.1186/s12931-019-1036-8 (PMC6444755; doi:10.1186/s12931-019-1036-8)

Figure E1. QQ plot of resting heart rate
LDSC intercept=1.1256, SE=0.0502

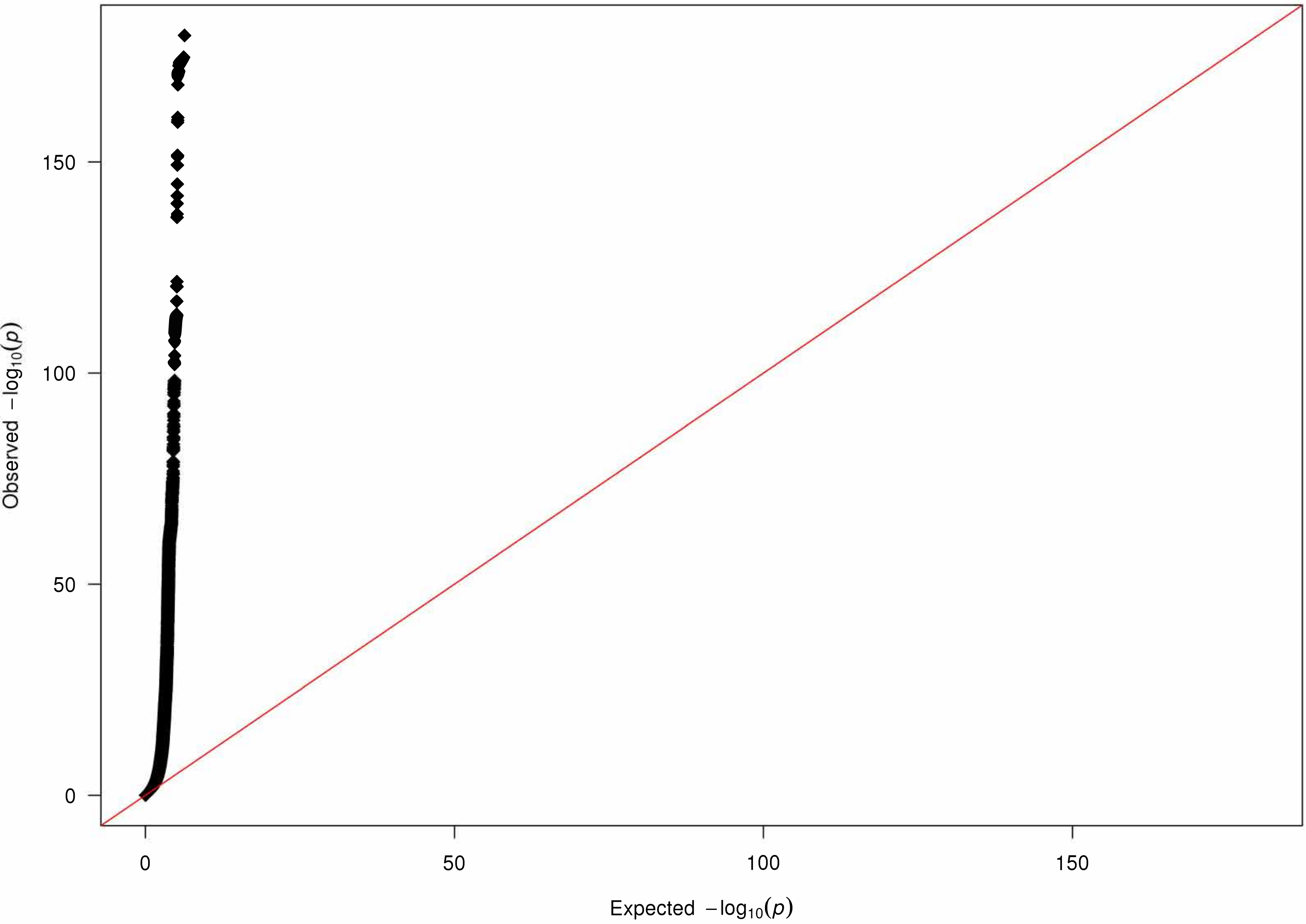

Figure E2. QQ plot of high blood pressure
LDSC intercept=1.1061, SE=0.0244

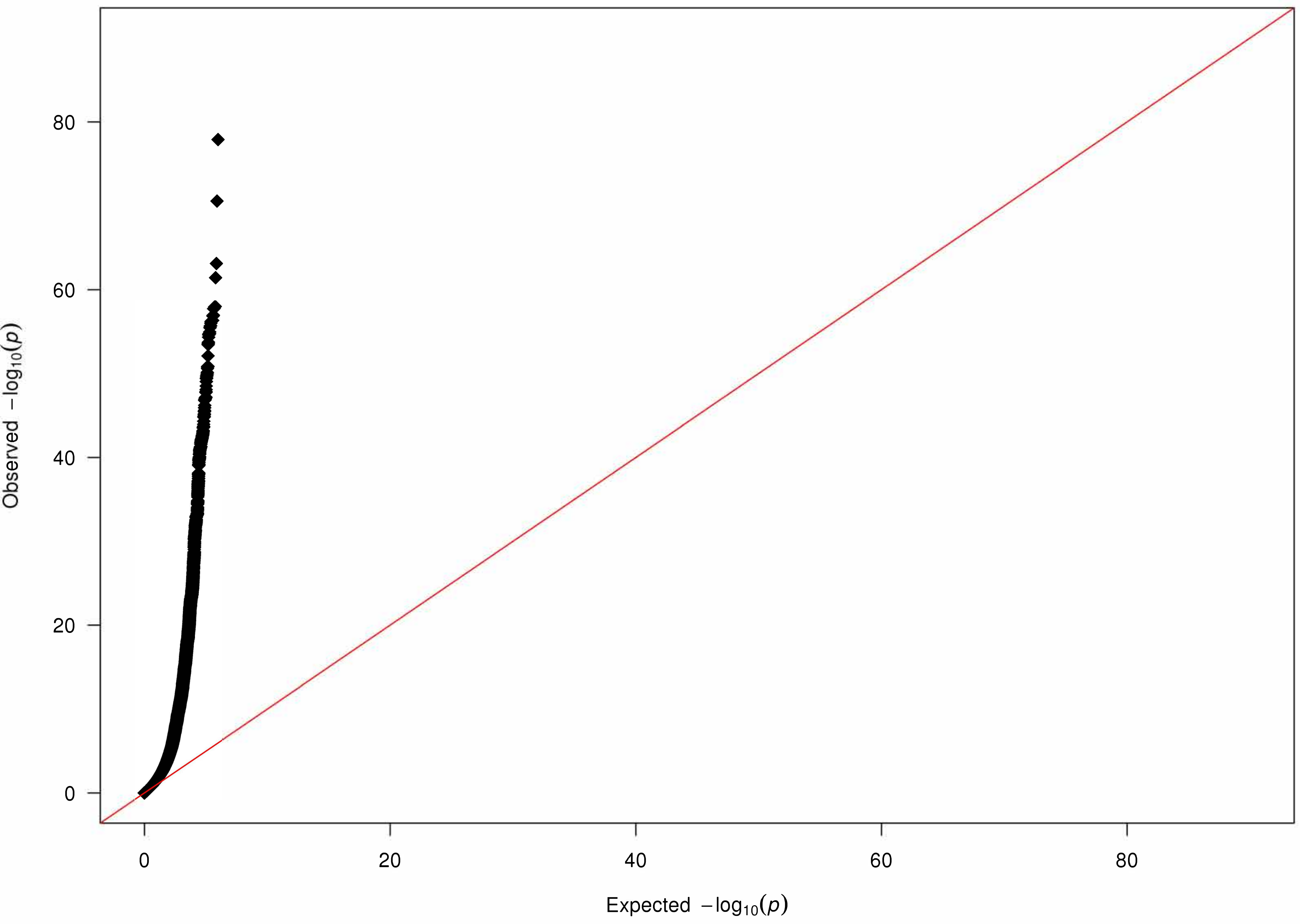

Figure E3. Genetic Correlation between COPD and Cardiac Traits by Functional Category

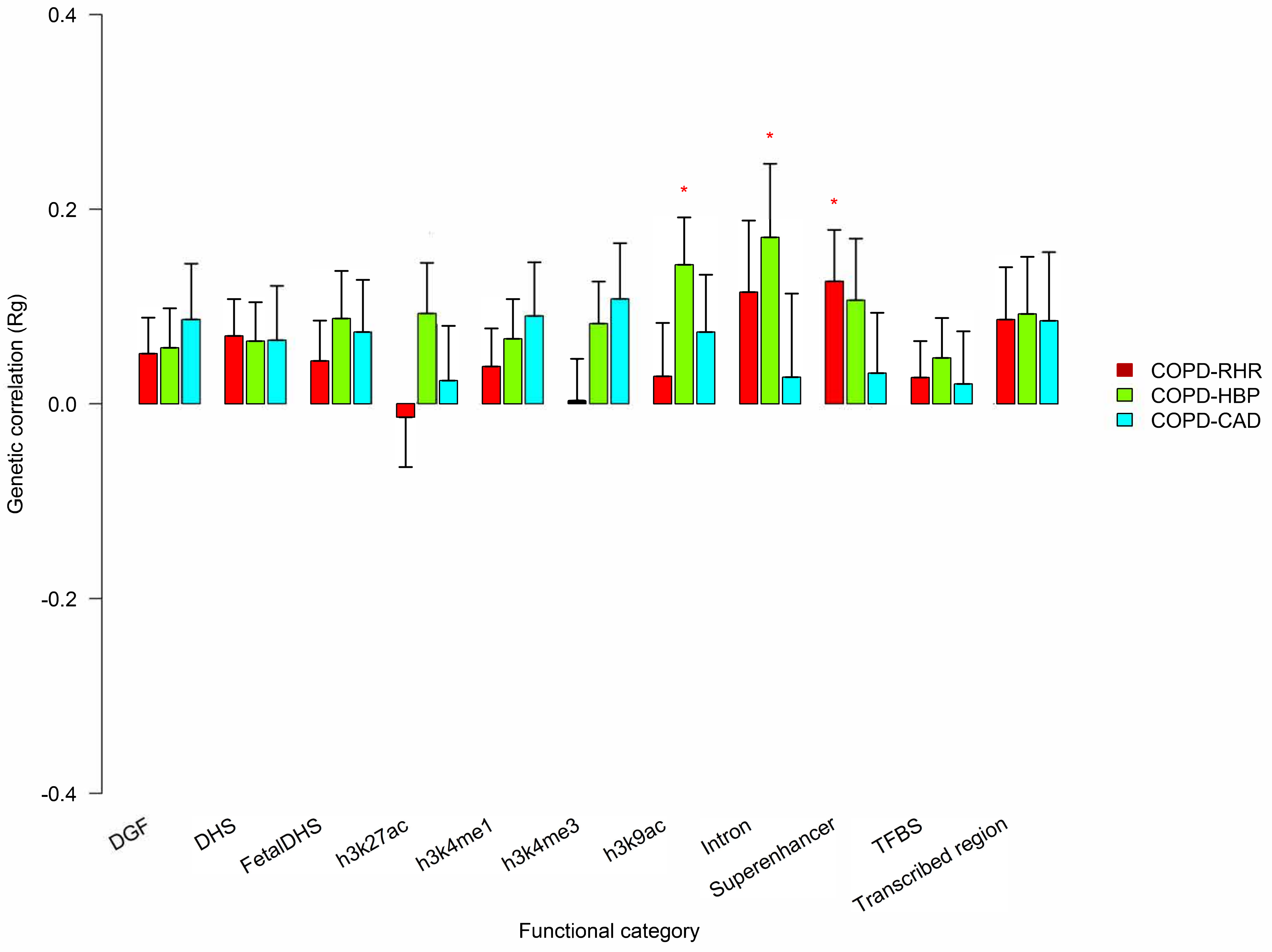

Supplement: Supplementary file 3 — Figure S1. QQ plot of resting heart rate. Figure S2. QQ plot of high blood pressure. Figure S3. Genetic Correlation between COPD and Cardiac Traits by Functional Category. (PDF 360 kb) [file 12931_2019_1036_MOESM3_ESM.pdf]
